# Meta-Analysis of the Alzheimer’s Disease Human Brain Transcriptome and Functional Dissection in Mouse Models

**DOI:** 10.1016/j.celrep.2020.107908

**Published:** 2020-07-14

**Authors:** Ying-Wooi Wan, Rami Al-Ouran, Carl G. Mangleburg, Thanneer M. Perumal, Tom V. Lee, Katherine Allison, Vivek Swarup, Cory C. Funk, Chris Gaiteri, Mariet Allen, Minghui Wang, Sarah M. Neuner, Catherine C. Kaczorowski, Vivek M. Philip, Gareth R. Howell, Heidi Martini-Stoica, Hui Zheng, Hongkang Mei, Xiaoyan Zhong, Jungwoo Wren Kim, Valina L. Dawson, Ted M. Dawson, Ping-Chieh Pao, Li-Huei Tsai, Jean-Vianney Haure-Mirande, Michelle E. Ehrlich, Paramita Chakrabarty, Yona Levites, Xue Wang, Eric B. Dammer, Gyan Srivastava, Sumit Mukherjee, Solveig K. Sieberts, Larsson Omberg, Kristen D. Dang, James A. Eddy, Phil Snyder, Yooree Chae, Sandeep Amberkar, Wenbin Wei, Winston Hide, Christoph Preuss, Ayla Ergun, Phillip J. Ebert, David C. Airey, Sara Mostafavi, Lei Yu, Hans-Ulrich Klein, Accelerating Medicines Partnership-Alzheimer’s Disease Consortium, Gregory W. Carter, David A. Collier, Todd E. Golde, Allan I. Levey, David A. Bennett, Karol Estrada, T. Matthew Townsend, Bin Zhang, Eric Schadt, Philip L. De Jager, Nathan D. Price, Nilüfer Ertekin-Taner, Zhandong Liu, Joshua M. Shulman, Lara M. Mangravite, Benjamin A. Logsdon

**Affiliations:** 1 Department of Molecular and Human Genetics, Baylor College of Medicine, Houston, TX 77030, USA; 2 Jan and Dan Duncan Neurologic Research Institute, Texas Children’s Hospital, Houston, TX 77030, USA; 3 Department of Pediatrics, Baylor College of Medicine, Houston, TX 77030, USA; 4 Sage Bionetworks, Seattle, WA 98121, USA; 5 Department of Neurology, Baylor College of Medicine, Houston, TX 77030, USA; 6 Department of Neurobiology and Behavior, University of California, Irvine, CA 92697, USA; 7 Institute for Systems Biology, Seattle, WA 98109, USA; 8 Rush Alzheimer’s Disease Center, Rush University Medical Center, Chicago, IL, USA; 9 Mayo Clinic, Department of Neuroscience, Jacksonville, FL 32224, USA; 10 Department of Genetics and Genomic Sciences, Mount Sinai Center for Transformative Disease Modeling, Icahn Institute for Data Science and Genomic Technology, Icahn School of Medicine at Mount Sinai, One Gustave L. Levy Place, New York, NY 10029, USA; 11 The Jackson Laboratory, Bar Harbor, ME 04609, USA; 12 Huffington Center on Aging, Baylor College of Medicine, Houston, TX 77030, USA; 13 Neuroscience DPU, Shanghai R&D, GlaxoSmithKline, Shanghai, China; 14 Neuroregeneration and Stem Cell Programs, Institute for Cell Engineering, Johns Hopkins University School of Medicine, Baltimore, MD 21205, USA; 15 Department of Physiology, Johns Hopkins University School of Medicine, Baltimore, MD 21205, USA; 16 Solomon H. Snyder Department of Neuroscience, Johns Hopkins University School of Medicine, Baltimore, MD 21205, USA; 17 Adrienne Helis Malvin & Diana Helis Henry Medical Research Foundations, New Orleans, LA 70130, USA; 18 Department of Pharmacology and Molecular Sciences, Johns Hopkins University School of Medicine, Baltimore, MD 21205, USA; 19 Department of Neurology, Johns Hopkins University School of Medicine, Baltimore, MD 21205, USA; 20 The Picower Institute for Learning and Memory, Department of Brain and Cognitive Sciences, Massachusetts Institute of Technology, Cambridge, MA 02139, USA; 21 Broad Institute of Harvard University and the Massachusetts Institute of Technology, Cambridge, MA 02139, USA; 22 Departments of Neurology and Pediatrics, Icahn School of Medicine at Mount Sinai, One Gustave L. Levy Place, New York, NY 10029, USA; 23 Evelyn F. and William L. McKnight Brain Institute, Center for Translational Research in Neurodegenerative Disease, Department of Neuroscience, University of Florida, Gainesville, FL 32610, USA; 24 Mayo Clinic, Department of Health Sciences Research, Jacksonville, FL 32224, USA; 25 Department of Biochemistry, Emory University School of Medicine, Atlanta, GA 30322, USA; 26 Data & Statistical Sciences, AbbVie, Cambridge, MA, USA; 27 Sheffield Institute of Translational Neuroscience, University of Sheffield, Sheffield, S10 2HQ, UK; 28 Molecular Oncology Lab, Cancer Research UK – Manchester Institute, The University of Manchester, Manchester, SK10 4TG, UK; 29 Department of Biosciences, Durham University, Durham, DH1 3LE, UK; 30 Beth Israel Deaconess Medical Center and Harvard Medical School, Boston, MA, USA; 31 Translational Genome Sciences, Biogen, Cambridge, MA, USA; 32 Eli Lilly & Company, Lilly Corporate Center, Indianapolis, IN 46285, USA; 33 University of British Columbia, Vancouver, BC, Canada; 34 Center for Translational & Computational Neuroimmunology, Department of Neurology and Taub Institute for the Study of Alzheimer’s Disease and the Aging Brain, Columbia University Irving Medical Center, New York, NY 10032, USA; 35 Cell Circuits Program, Broad Institute, Cambridge, MA 02142, USA; 36 Full consortium author list available at http://doi.org/10.7303/syn17114455; 37 Eli Lilly & Company, Erl Wood Manor, Sunninghill Road, Windlesham, Surrey, GU20 6PH, UK; 38 Department of Neurology, Emory University School of Medicine, Atlanta, GA 30322, USA; 39 Foundation Neuroscience Center, AbbVie, Cambridge, MA, USA; 40 Mayo Clinic, Department of Neurology, Jacksonville, FL 32224, USA; 41 Department of Neuroscience, Baylor College of Medicine, Houston, TX 77030, USA; 42 Present address: Department of Molecular and Cell Biology, University of California, Berkeley, Berkeley, CA 94720, USA; 43 These authors contributed equally; 44 These authors contributed equally; 45 Senior author; 46 Lead Contact

## Abstract

We present a consensus atlas of the human brain transcriptome in Alzheimer’s disease (AD), based on meta-analysis of differential gene expression in 2,114 postmortem samples. We discover 30 brain coexpression modules from seven regions as the major source of AD transcriptional perturbations. We next examine overlap with 251 brain differentially expressed gene sets from mouse models of AD and other neurodegenerative disorders. Human-mouse overlaps highlight responses to amyloid versus tau pathology and reveal age- and sex-dependent expression signatures for disease progression. Human coexpression modules enriched for neuronal and/or microglial genes broadly overlap with mouse models of AD, Huntington’s disease, amyotrophic lateral sclerosis, and aging. Other human coexpression modules, including those implicated in proteostasis, are not activated in AD models but rather following other, unexpected genetic manipulations. Our results comprise a cross-species resource, highlighting transcriptional networks altered by human brain pathophysiology and identifying correspondences with mouse models for AD preclinical studies.

## In Brief

Wan et al. develop a consensus atlas of the human brain transcriptome in Alzheimer’s disease, based on 2,114 postmortem samples, and examine overlap with 251 expression signatures from mouse brain RNA sequencing experiments, including many neurodegenerative disease models.

## Graphical Abstract

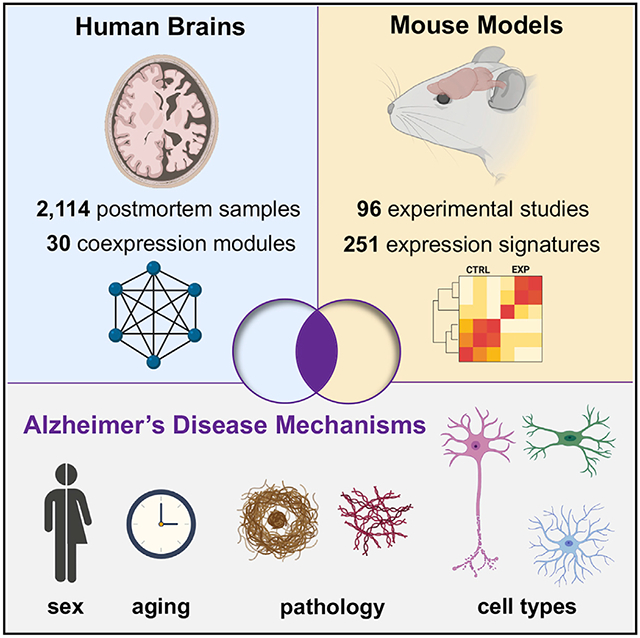

## INTRODUCTION

Alzheimer’s disease (AD) is a progressive and incurable neurodegenerative disorder, with rapidly increasing prevalence due to population aging and no disease-modifying interventions (Scheltens et al., 2016). At autopsy, AD is characterized by extracellular amyloid plaques and intraneuronal neurofibrillary tangles, composed of misfolded and aggregated amyloid-beta (Aβ) peptide and microtubule-associated protein tau (MAPT or tau), respectively. The mechanisms by which this AD pathologic cascade is triggered and propagated remain incompletely defined and are likely influenced by heterogeneous and dynamic perturbations involving multiple biological pathways. Besides synaptic and neuronal loss, AD pathology is also accompanied by changes in astrocytic and microglial cells, and human genome-wide association studies (GWASs) support a causal role for immune mechanisms (Jansen et al., 2019; Kunkle et al., 2019; Raj et al., 2014). Results from RNA sequencing (RNA-seq) of human postmortem brain tissue further reinforce the importance of microglial and inflammatory mechanisms in AD pathogenesis, among other pathways (Allen et al., 2018; Conway et al., 2018; McKenzie et al., 2017; Patrick et al., 2017; Wang et al., 2016, 2019; Zhang et al., 2013).

Compared with transcriptome profiles in human brains, mouse models allow controlled experimental manipulations that can be used to establish causation, isolate the effects of specific molecular lesions, and investigate for dynamic, age-dependent changes. A large number of AD mouse models have been extensively characterized, and these systems have contributed to our understanding of disease pathogenesis (Götz et al., 2018; Jankowsky and Zheng, 2017; LaFerla and Green, 2012). The most widely used transgenic models express mutant forms of the amyloid precursor protein (APP) gene, with or without presenilin-1/2 (*PSEN1/2*), which are associated with autosomal-dominant, early-onset AD, or, alternatively, *MAPT* mutations, which cause familial frontotemporal dementia (FTD). These models recapitulate features of AD neuropathology, including plaques or tangles, along with variable degree of neuronal dysfunction or loss and progressive behavioral impairment (Ballatore et al., 2007; Esquerda-Canals et al., 2017). More recently, gene expression profiling, including RNA-seq, has been applied to elucidate brain transcriptome signatures in mouse models. These investigations have similarly highlighted the potential importance of immune or inflammatory and neuronal or synaptic changes (Boisvert et al., 2018; Castanho et al., 2020; Cummings et al., 2015; Gjoneska et al., 2015; Matarin et al., 2015; Rothman et al., 2018; Stephenson et al., 2018; Swartzlander et al., 2018). Although some studies have identified selected overlaps in expression changes between AD mouse models and human brains (Bennett et al., 2018; Castillo et al., 2017; Mostafavi et al., 2018; Neuner et al., 2019; Rojo et al., 2017), other studies have questioned the overall degree of conservation (Burns et al., 2015; Galatro et al., 2017; Hargis and Blalock, 2017).

The Accelerating Medicines Partnership-Alzheimer’s Disease (AMP-AD) Consortium has generated RNA-seq profiles from more than 1,200 human brains and is applying systems biology approaches toward the goal of elucidating AD mechanisms and potential therapeutic targets. Here, we perform a meta-analysis including all available AMP-AD RNA-seq datasets and systematically define correspondences between gene expression changes associated with AD in human brains and those caused by controlled experimental manipulations in mouse models, including studies relevant to AD, other neurologic disorders, brain health, and aging. Our results constitute a powerful reference of transcriptome perturbations in AD, highlight sex as an important driver, and identify strengths and limitations among currently available mouse models.

## RESULTS

### A Consensus Atlas of AD-Associated Transcriptional Modules from Human Brain

Because transcript abundance is highly influenced by multiple sources of technical and biological variation, we first evaluated the consistency and robustness of findings across three independent human brain transcriptome studies (ROSMAP [Religious Orders Study and the Memory and Aging Project], MSSM [Mount Sinai School of Medicine], and Mayo), consisting in total of 2,114 samples from 1,234 subjects (Table S1). Human postmortem brain RNA-seq data were obtained from seven distinct regions: dorsolateral prefrontal cortex (DLPFC), temporal cortex (TCX), inferior frontal gyrus (IFG), superior temporal gyrus (STG), frontal pole (FP), parahippocampal gyrus (PHG), and cerebellum (CBE). We applied a stringent definition of AD (n = 478 cases), requiring both a clinical diagnosis and neuropathologic confirmation (Table 1). All control brains (n = 300) were devoid of significant AD pathology, and in two of the three cohorts, control subjects were clinically confirmed as non-demented. To identify transcriptional modules associated with AD, we integrated differential expression and network coexpression analyses. First, we performed both random- and fixed-effects meta-analyses (DerSimonian and Laird, 1986; Mantel and Haenszel, 1959) of differential expression across the brain regions, defining as many as 2,355 upregulated and 2,130 downregulated transcripts in AD brains (Table 2; Supplemental Information). Next, we performed coexpression analysis using five distinct algorithms (see STAR Methods), generating 2,978 brain region-specific coexpression modules (CBE, n = 498; DLPFC, n = 450; FP, n = 393; IFG, n = 429; PHG, n = 370; STG, n = 336; TCX, n = 502) (Supplemental Information). As expected, similar overall coexpression was observed in each brain region dataset independent of the algorithm used, based on gene overlap among modules (p_adj_ < 0.05, Fisher’s exact test). In order to define consensus modules that were differentially expressed in AD, we first restricted our analysis to individual modules that were significantly enriched for the differentially expressed genes (DEGs) from the meta-analysis (p_adj_ < 0.05, Fisher’s exact test from either the fixed- or random-effects model results). We next applied graph clustering (Pons and Latapy, 2005) to define brain region-specific consensus modules based on patterns of shared module gene membership. This analysis culminated in 30 AD-associated modules across the seven tissue types (CBE, n = 4; DLPFC, n = 4; FP, n = 4; IFG, n = 4; PHG, n = 5; STG, n = 4; TCX, n = 5), with module sizes ranging from 504 to 4,673 genes (mean 2,090) (Figures S1A–S1E).

**Figure 1.**
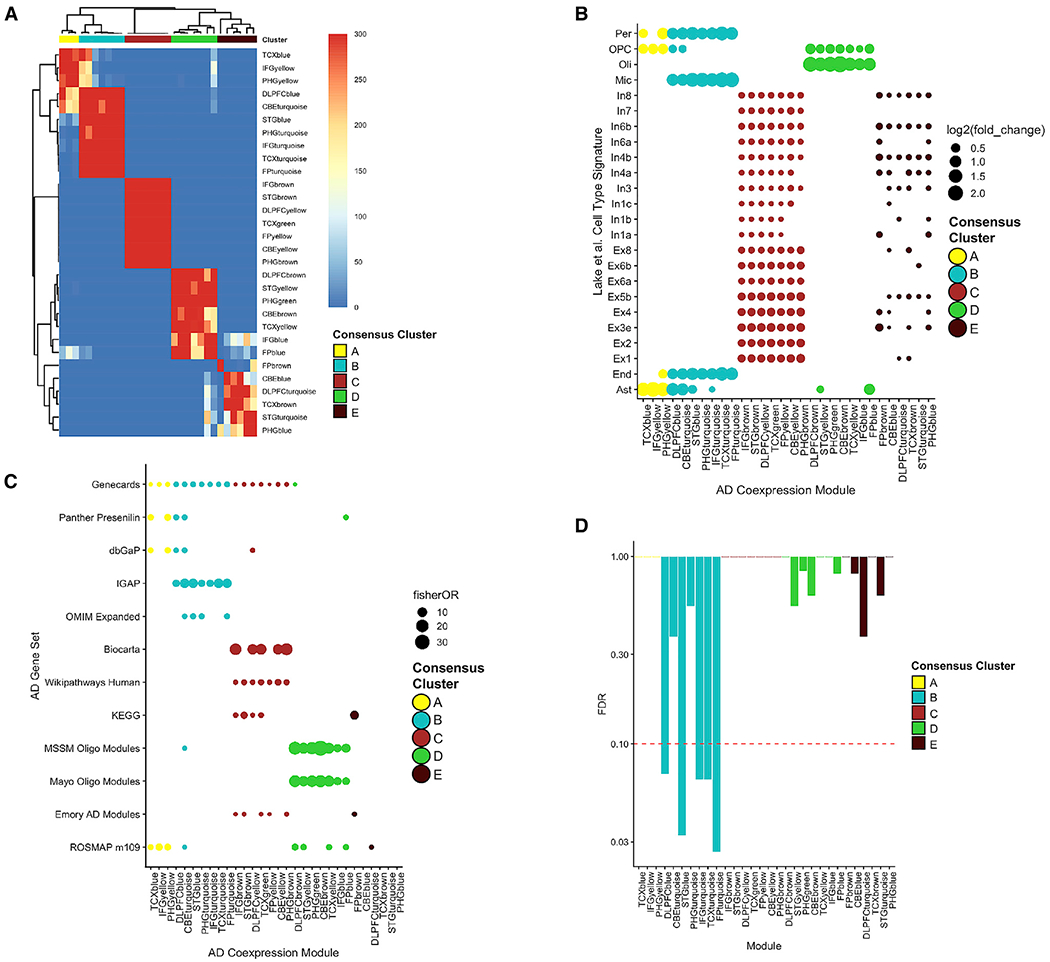
Human Consensus RNA-Seq Coexpression Modules (A) Gene set overlap (Fisher’s exact test p value) was examined among 30 AD-associated coexpression modules, highlighting five consensus clusters (A, B, C, D, and E). (B) Coexpression modules were evaluated using expression-weighted cell type enrichment analysis based on human single-cell RNA-seq. (C) Coexpression modules were examined for overlap (Fisher’s exact test) with curated AD gene sets from GeneCards, Panther, the Database of Genotypes and Phenotypes (dbGaP), IGAP, Online Mendelian Inheritance in Man (OMIM), Biocarta, Wikipathways, and the Kyoto Encyclopedia of Genes and Genomes (KEGG). We also evaluated overlap with coexpression modules derived from the constituent cohorts, including oligodendroglial modules identified by Mayo and MSSM, module 109 from ROSMAP, and an RNA-binding protein rich module from Emory. (D) Coexpression module enrichment for AD susceptibility gene candidates from GWAS, based on MAGMA. Consensus cluster B modules appear strongly enriched for AD risk loci.

Evaluation across tissues demonstrated that these 30 modules fell into five distinct “consensus clusters” that were highly preserved across study and tissue type and demonstrated minimal overlap (henceforth denoted clusters A–E) (Figure 1A; Figure S1). Indeed, further analysis of the consensus clusters revealed significant conservation across brain regions, with clusters A, B, and C each demonstrating a mean gene overlap of 83% (SE 0.035) among the constituent modules (i.e., proportion of genes that are shared at least once across modules in the cluster). Consensus clusters D and E had a slightly lower mean overlap of 77% (SE 0.030) in gene set membership among the constituent modules. Compared with the 30 AD-associated modules, other non-associated coexpression modules were less conserved across brain regions (see Figure S2A; Supplemental Information). Modules also included a minority of “private” genes (not seen in other modules within clusters), identifying potential unique regional signatures across the brain. Coexpression modules derived from CBE showed the highest number of private genes, potentially consistent with its distinct cellular architecture (Figure S1).

We next evaluated consensus clusters for enrichment of cell type-specific gene signatures (Figure 1B), using expression-weighted cell type enrichment analysis (Skene and Grant, 2016) and a human single-cell RNA-seq reference dataset (Lake et al., 2018). Consensus clusters were enriched for astrocytes and pericytes (clusters A and B), endothelial cells (B), microglia (B), neurons (C and E), oligodendroglia (D), and oligodendroglial precursors (A and D). We obtained consistent results for overlaps using Fisher’s exact test (Figure S2B) and leveraging a complementary cell type reference dataset from mouse brains (Zhang et al., 2014) (Figure S2C). Despite the significant overlaps, we note that cell type-specific signatures constitute a minority of genes within each large cluster. Clusters A–E are also enriched for a diversity of biological processes (Figure 1C; Supplemental Information). Notably, consensus cluster E was enriched for multiple gene sets involved in the regulation of proteostasis, including both the heat shock and unfolded protein responses (Table S2). Last, based on linkage disequilibrium (LD) score regression method in MAGMA (de Leeuw et al., 2015), multiple cluster B modules were significantly enriched for AD GWAS loci (Kunkle et al., 2019) (Figure 1D).

As expected, several of the consensus clusters generated by our meta-analysis recapitulate molecular networks reported in prior analyses restricted to the constituent datasets. For example, independent analyses of RNA-seq from the Mayo and MSSM cohorts previously identified AD-associated coexpression modules enriched for oligodendrocyte expression signatures (Allen et al., 2018; McKenzie et al., 2017), and these gene sets significantly overlapped with consensus cluster D (Figure 1C). We also examined a coexpression module (m109) significantly associated with both AD pathology and rate of cognitive decline based on an independent analysis of RNA-seq data from the ROSMAP cohort (Mostafavi et al., 2018). Interestingly, m109 showed significant overlap with four of the five consensus clusters (Figure 1C), suggesting that it may represent a coordinated brain transcriptional response involving multiple cell types. Last, we examined an AD-associated coexpression module enriched for RNA-binding proteins that was previously nominated from human brain proteomics (Johnson et al., 2019). This gene set significantly overlapped with cluster C, suggesting that it may be coregulated with neuronal genes.

### Overlaps with AD Mouse Models

**Figure 2.**
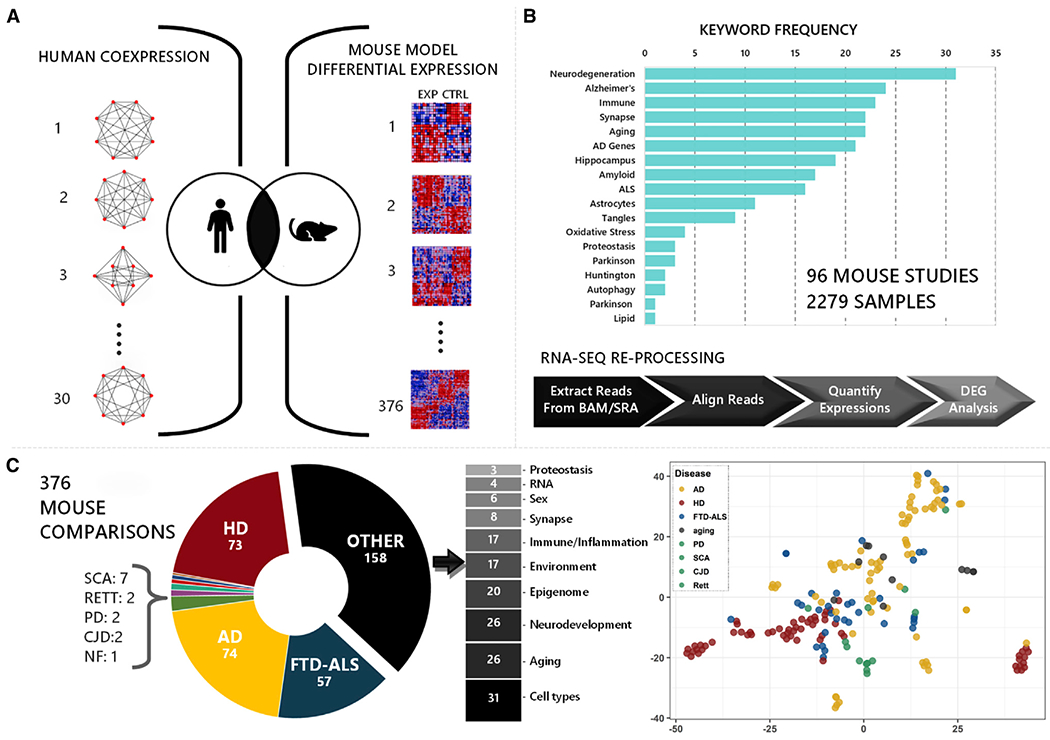
Cross-Species Study Design and Data (A) Analytic design for examining overlaps between 30 Alzheimer’s disease (AD)-associated human coexpression modules and differentially expressed gene sets from 376 experimental comparisons in mouse models. (B) Ninety-six mouse studies were selected based on relevance to AD and other neurodegenerative disorders. Distribution of keywords is shown among all studies. RNA-seq was reprocessed using a standard pipeline. See Table S3 for details on all included mouse studies. (C) The differentially expressed gene sets represent mouse models of AD, Huntington’s disease (HD), frontotemporal dementia-amyotrophic lateral sclerosis (FTD-ALS), spinocerebellar ataxia 1 (SCA1), Rett syndrome (RETT), Parkinson’s disease (PD), Creutzfeldt-Jakob disease (CJD), neurofibromatosis (NF), or other neurodegenerative mechanisms. A t-distributed stochastic neighbor embedding (t-SNE) plot including all mouse differential expression signatures highlight both disease-specific and overlapping features among heterogeneous neurodegenerative models. See also Figure S3.

**Figure 3.**
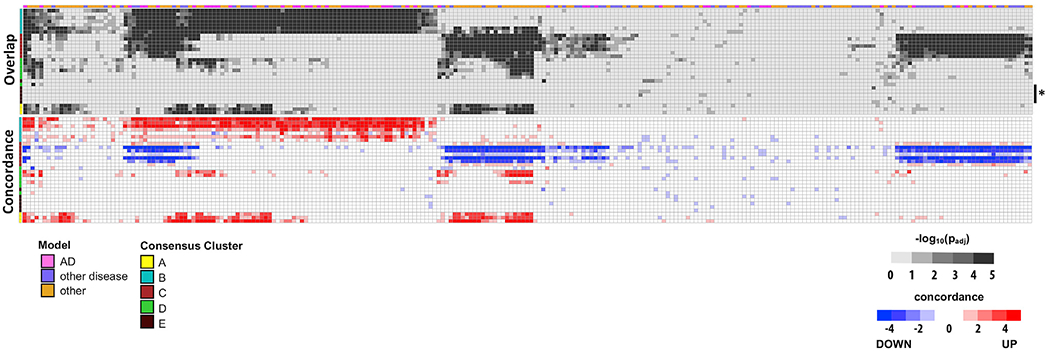
Overview of Human-Mouse Overlaps and Concordance Heatmaps show overlap (top) and concordance (bottom) among 30 human coexpression modules (rows) and 251 sets of differentially expressed genes (DEGs; columns) from mouse model comparisons. The average sample size is 8.4 (range 4–28 total samples). Mouse-human overlap significance, calculated using the hypergeometric test, is represented in grayscale (−log_10_[p_adj_]). Direction (red/blue) and extent of concordance (intensity) for gene expression changes are also indicated (bottom). The color bar at the top annotates all DEGs based on whether they derive from Alzheimer’s disease (AD) models (pink), other neurodegenerative models (purple), or other experimental manipulations potentially relevant to AD mechanisms (orange). The color barat left denotes cluster membership (A–E). Cluster E modules (brown, asterisk) show sparse overlap with AD mouse model DEGs. See Tables S4, S5, and S6 for details on all experimental comparisons and comprehensive results.

To enable cross-species comparisons, we uniformly reprocessed brain RNA-seq data from 96 distinct mouse studies relevant to AD, other neurodegenerative disorders, aging, and related mechanisms (Figure 2; Table S3). We next curated 376 unique experimental comparisons (genetic manipulation versus control condition). After applying quality control filters (gene membership > 10, fold change > 1.2, false discovery rate < 1%; see also STAR Methods), we define 251 sets of significant DEGs comprising brain “gene expression signatures” (mean 1,385 genes, range 10–12,393) characteristic of each mouse model comparison (Table S4; Supplemental Information). These expression profiles encompass 25,181 unique transcripts among 52,873 total in the mouse transcriptome. The curated expression signatures capture a combination of disease-specific and overlapping features characteristic of the heterogeneous mouse models included in this study, on the basis of t-weighted stochastic neighbor embedding (t-SNE) plots (Figure 2C). Even among mouse models within the same disease category, the overall extent of gene overlap among the corresponding sets of DEGs was modest (Figure S3A); therefore, the included comparisons sample a wide spectrum of brain transcriptional responses. We next evaluated the overlap between each mouse expression signature and the 30 human AD-associated brain consensus coexpression modules defined by our meta-analysis. Overall, we detected 1,569 significant overlaps (p_adj_ < 0.01), with the majority (68%) of experimental comparisons having gene expression changes that corresponded to multiple human modules (mean 6 overlapping modules per mouse model DEG set) (Figure 3; Table S5). As expected, most mouse DEG sets showed consistent overlaps with human modules that fell within consensus clusters A–D. We next assessed whether the direction of gene expression changes in mouse and human brains was concordant, that is, whether transcriptional perturbations are phased in the same direction (both up- or downregulated). Indeed, the majority (77%) of overlapping gene expression signatures were concordant in direction across species (Figure 3). For example, cluster B genes were consistently upregulated in human AD brain transcriptomes and mouse AD models, whereas cluster C genes were predominantly downregulated. The exceptional discordant modules (e.g., FPturquoise and DLPFCyellow in clusters B and C, respectively) may highlight coregulated gene sets with greater temporal or regional fluctuations over the disease course. Last, 28 of 30 human coexpression modules significantly overlapped (p_adj_ < 0.01) with at least one mouse model expression signature. In sum, our results support broad conservation of gene regulatory systems in the mammalian brain, highlighting that many AD-associated brain expression patterns are recapitulated in mouse experimental models.

**Figure 4.**
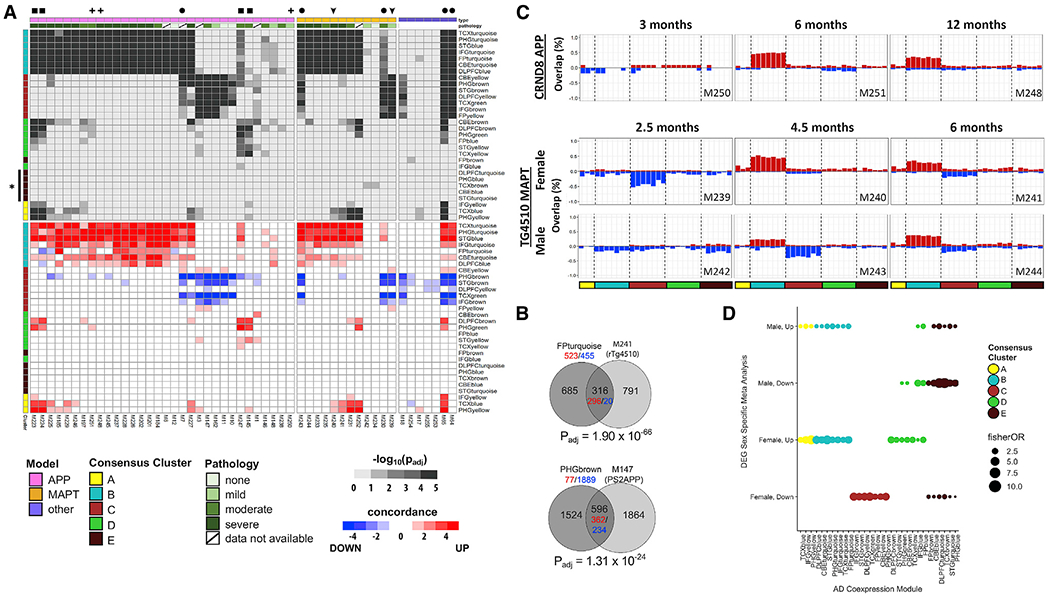
Human Coexpression Module Overlaps with AD Mouse Models (A) Heatmaps show overlap (top, hypergeometric test) and concordance (bottom) among human coexpression modules and sets of differentially expressed genes (DEGs) from Alzheimer’s disease (AD) mouse models. Mouse-human overlap significance, calculated using the hypergeometric test, is represented in grayscale (−log_10_[p_adj_]). The color bar at the top denotes APP (pink), MAPT (orange), or other (purple) model comparisons. The estimated pathologic burden (plaques/tangles and neuronal loss) in APP and MAPT models is also annotated in green. Cluster E modules (brown, asterisk) shows parse overlap with AD mouse models. Selected overlaps are denoted as follows: squares, APP models with oligodendrocyte-enriched module overlaps; cross-hatches, CRND8-APP models showing sustained activation of microglial modules from 6 months onward; arrowhead, transient activation of neuronal modules in TG4510-MAPT model preceding microglial module overlap; circles, co-activation of neuronal and microglial modules. See Tables S4 and S5 for details on all experimental comparisons, including sample sizes and genotypes, along with comprehensive results. See Figure S4 for duplicated panel including detailed model annotations. See Figure S6 for additional analysis of overlap specificity. (B) Representative overlaps of human modules with mouse DEGs. The hypergeometric test was applied to assess significance. Gene counts are noted in black, including for overlapping and non-overlapping regions. To assess concordance between human brains and mouse models, gene counts are shown, noting increased (red) or decreased expression (blue), including for the human coexpression module and the overlapping mouse gene set. M147 was derived from Srinivasan et al. (2016). (C) Mouse model overlaps highlight age- and sex-dependent changes. Increasing (red) or decreasing (blue) gene expression and magnitude of changes shown as overlap (%) between the mouse DEG set and module. Cell type module clusters are denoted by colors at panel bottom, as in (A). (D) Enrichment of human sex-specific DEGs from random-effects meta-analysis among coexpression modules. Consensus cluster C shows downregulation in AD among females.

Our reprocessed dataset includes 53 expression signatures from 12 distinct AD transgenic models, including multiple *APP* and *MAPT* transgenic strains (Figure 4A). Human microglial- and neuronal-enriched clusters (B and C, respectively) strongly overlapped with expression signatures from mouse models of AD (Figures 4A–4C). For example, module FPturquoise from cluster B significantly overlapped (p_adj_ <1 × 10^−5^) with the majority of expression signatures from both *APP* (66%) and *MAPT* (67%) mouse models. Although overlaps with cluster C modules (e.g., PHGbrown) were more restricted, significant overlaps were still observed in a substantial proportion of AD mouse models (22% and 25% of *APP* and *MAPT* models, respectively). In most cases, attenuation of cluster C gene expression and activation of cluster B appeared to be mutually exclusive (Figures 4A and 4C). Interestingly, coincident changes in both cluster B and cluster C module genes (Figure 4A, circles) were characteristic of selected *MAPT* strains and were also seen in the *CDK5-P25* mouse, which similarly develops extensive tau pathology and brain atrophy (Cruz et al., 2003). This overlap pattern was only rarely seen among the *APP* models (1 of 34 DEG sets). Potentially consistent with late microgliosis, *APP* and *MAPT* mouse expression signatures demonstrated stronger overlaps with cluster B modules at more advanced stages of brain pathology (Figure 4A). Reciprocally, the human cluster C modules overlapped expression signatures from mouse models with comparatively mild pathologic burden, suggestive of early-stage synaptic and neuronal dysfunction. RNA-seq profiles generated from multiple aged time points allow examination of dynamic changes in human overlap patterns for selected AD models (Figure 4C). For example, in the TgCRND8 *APP* transgenic mouse, cluster B module activation was seen by 6 months, and this signature was sustained at 12 months. In contrast, in the rTg4510 *MAPT* mouse model, transient gene expression changes overlapping cluster C human modules either preceded or accompanied the appearance of cluster B expression signatures (M239–M244; Figure 4A, arrowheads, and Figure 4C, bottom). Compared with cluster B and C modules, overlaps between AD mouse model expression signatures and clusters A, D, and/or E were more sparsely detected (Figure 4A). For example, TCXyellow and STGyellow–two cluster D modules similarly enriched for oligodendroglial expression signatures including genes with roles in sphingolipid metabolism–showed selective overlap with aged *APP* transgenic models (e.g., TgCRND8 [M247], 5xFAD [M223, M224], and PS2APP [M145]) (Figure 4A, squares; Figure S4).

### Sex-Specific Expression Signatures in Humans and Mice

Prior observations in AD mouse models (Jiao et al., 2016) and human epidemiology (Altmann et al., 2014; Li and Singh, 2014; Mayeux and Stern, 2012) have implicated sex as a modifier of AD manifestation and progression. In selected cases, our cross-species analyses provided consistent evidence of a sex-by-age interaction. Compared with males, female rTg4510 mice demonstrated overlaps with human coexpression modules consistent with accelerated progression in brain transcriptional changes (Figure 4C, bottom). Specifically, compared with male mice, for which downregulated genes (M242) do not show overlaps with cluster C modules until 4.5 months, genotype-matched female mice (M239) exhibited similar changes at 2.5 months. Furthermore, beginning at 4.5 months, upregulated genes from females also showed a strong overlap with cluster B modules. These results suggest that sex may modify age- and AD pathology-dependent progression of brain expression changes.

We next repeated our differential expression meta-analysis of human RNA-seq data and stratified by sex (Table 1). Strikingly, we observed ~2.5 times more DEGs in women than in men, consistent with other recently published work (Mathys et al., 2019). Sex-specific DEGs also showed evidence of heterogeneous overlaps among the coexpression modules (Figure 4D). For example, clusters C and D were significantly and selectively enriched for female-specific DEGs. Although clusters A and B overlapped with DEGs from either sex, approximately 3-fold increased enrichment was seen for female DEGs compared with male DEGs. In contrast, male-specific DEGs exhibited preferential overlapping with cluster E. Specifically, upregulated transcripts from male subjects selectively overlapped cluster E modules, whereas downregulated cluster E transcripts showed stronger enrichment for female DEGs. Overall, results from both human brains and mouse models suggest that sex has a strong influence on brain gene expression changes in AD.

### Overlaps with Aging and Other Disease Expression Signatures

**Figure 5.**
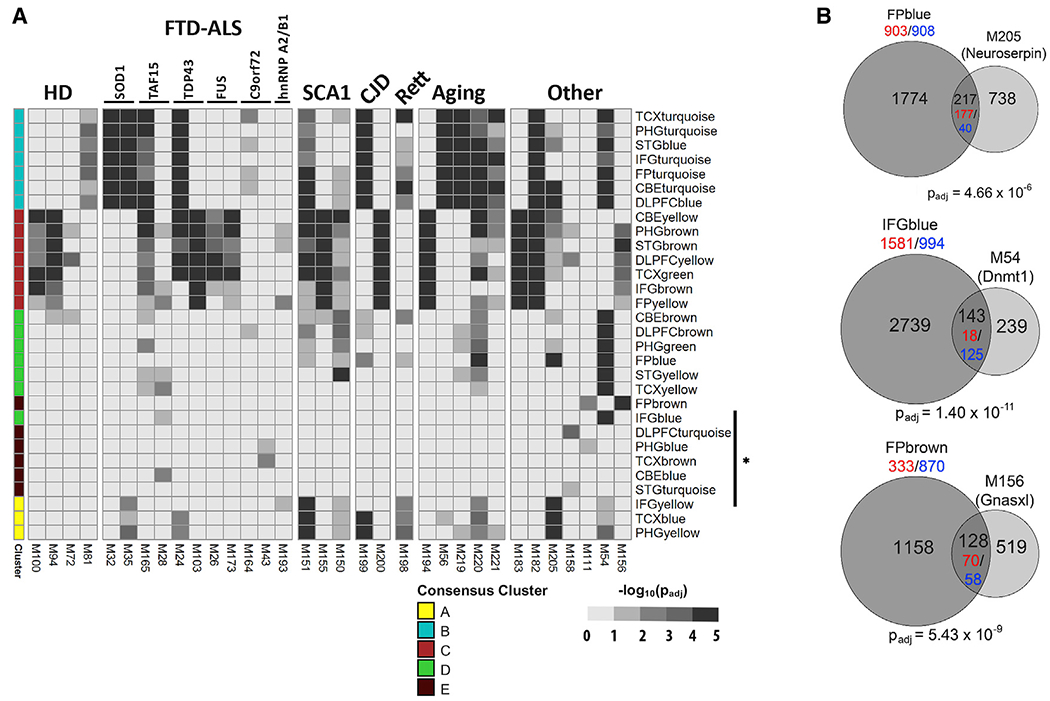
Overlaps with Other Mouse Models (A) Heatmaps show overlap among human coexpression modules and sets of differentially expressed genes (DEGs) from mouse models, including pure aging, neurodegenerative disorders, and other experimental manipulations. HD, Huntington’s disease; FTD-ALS, frontotemporal dementia-amyotrophic lateral sclerosis; SCA1, spinocerebellar ataxia 1; CJD, Creutzfeldt-Jakob disease. Mouse-human overlap significance, calculated using the hypergeometric test, is represented in grayscale (−log_10_[p_adj_]). Overlaps between HD model expression signatures (Langfelder et al., 2016) and neuronal gene-enriched human coexpression modules recapitulate polyglutamine length (M100, Q92 versus M94, Q175) and brain region dependence (M100/M94, striatum versus M72/M81, cortex). Other manipulations generate signatures similar to AD models, including *PTCH1* knockout (M183) (Ung et al., 2018), *nmf205* (M182) (Ishimura et al., 2016), and *neuroserpin* mutant (M205) (Guadagno et al., 2017). Modules poorly enriched for cell type signatures (asterisk, right) show selected overlaps with FTD-ALS models (M28, M43; Ibrahim et al., 2013, and Lagier-Tourenne et al., 2013, respectively) and other, unexpected genetic manipulations (M158, M111, M54, and M156; Vied et al., 2016, Maze et al., 2015, Narayanan et al., 2014, and Holmes et al., 2016, respectively). See Tables S5 and S6 for comprehensive results, including sample sizes for all comparisons. See Figure S5 for comprehensive heatmaps representing overlaps with HD, FTD-ALS, SCA1, and aging models. (B) Representative overlaps of human modules with mouse DEGs, as in Figure 4B. The hypergeometric test was applied to assess significance.

Numerous mechanistic parallels have been recognized between AD and other neurodegenerative disorders, including similar protein aggregate pathologies, proteostatic and oxidative stress, neuroinflammation, and the critical role of aging in disease risk and/or progression (Block and Hong, 2005; Bucciantini et al., 2002; Guo and Lee, 2014; Haass and Selkoe, 2007; Nixon, 2005; Ross and Poirier, 2004). We therefore examined for overlap between human AD coexpression modules and expression signatures from mouse experimental models for other neurodegenerative disorders, including Huntington’s disease (HD), FTD-amyotrophic lateral sclerosis (ALS), Parkinson’s disease, spinocerebellar ataxia 1 (SCA1), Creutzfeldt-Jakob disease (CJD) and Rett syndrome, along with data from aged, wild-type mice mouse strains (Figure 5; Figure S5). Similar to *APP/MAPT* transgenic mice, most other neurodegenerative disease mouse models strongly activated expression signatures overlapping with the cluster B and cluster C modules from human brain. For example, PHGbrown (cluster C) overlaps with mouse brain expression signatures from models of HD (M94, p_adj_ = 2.5 × 10^−10^), FTD-ALS (*FUS*) (M173, p_adj_ = 4.8 × 10^−6^), SCA1 (M155, p_adj_ = 1.5 × 10^−18^), and CJD (M200, p_adj_ = 6.0 × 10^−32^). Similarly, FPturquoise (cluster B) significantly overlaps with DEGs from models of FTD-ALS (*TDP43*) (M24, p_adj_ = 2.0 × 10^−42^), CJD (M199, p_adj_ = 3.7 × 10^−18^), and Rett syndrome (M198, p_adj_ = 3.1 × 10^−3^) and more selectively in HD (M81, p_adj_ = 1.1 × 10^−4^) and SCA1 (M151, p_adj_ = 5.3 × 10^−5^) models. Therefore, these signatures likely represent common brain transcriptome responses induced by diverse neurodegenerative triggers. Indeed, we found that genes implicated in immune biology and inflammation constitute those recurring most frequently among the 251 mouse gene expression signatures included in this study (Figure S3B), consistent with the overlaps seen for cluster B, which is significantly enriched for microglial expression signatures. Importantly, expression signatures from aged, wild-type mice also show significant overlaps with human coexpression modules from clusters B (M56, M219), C (M194), or both B and C concurrently (M220, M221) (Figure 5). For example, compared with 3 month controls, hippocampal tissue from 24-month-old mice (M56) exhibited expression signatures overlapping with cluster B module FPturquoise (p_adj_ = 2.9 × 10^−17^). Overall, our results suggest that these patterns may not be specific for neurodegenerative disease, but rather may accompany brain aging more generally. We next examined for any human coexpression modules with overlaps showing relative specificity for AD mouse models. Although none showed absolute specificity, we found that FPblue and TCXyellow, both from the oligodendroglial-enriched cluster D, are strongly activated in selected AD models (e.g., M145, M247, M223, M65 in Figure 4) but show comparatively sparse or weak overlap with expression signatures from the majority of other neurodegenerative disease models. Thus, these modules may encompass transcriptional programs that are preferentially activated by AD pathophysiology.

In order to further assess AD specificity of the consensus modules defined in our analysis, we examined pairwise overlaps with published RNA-seq coexpression modules among 700 human brains from subjects with neuropsychiatric disorders (autism, schizophrenia, bipolar disorder, depression, and alcoholism) (Gandal et al., 2018). Interestingly, virtually all cluster D modules failed to overlap with modules showing significant associations with neuropsychiatric disorders (Figure S6A) and were therefore “AD specific.” Similar but less extreme evidence for specificity was seen for cluster E modules. In contrast, AD consensus clusters B and C strongly overlapped with several neuropsychiatric coexpression modules similarly enriched for microglial and neuronal expression signatures, respectively. For example, module CD11, which was increased in autism, overlapped significantly with AD cluster B module FPturquoise (p_adj_ = 1.3 × 10^−136^) (Figure S6B). As expected, the genes in CD11 overlapped with DEGs from multiple mouse models (M241, p_adj_ = 5.4 × 10^−29^; M24, p_adj_ = 5.3 × 10^−12^; M219, p_adj_ = 8.1 × 10^−12^), consistent with overlaps seen for consensus cluster B. Thus, rather than representing specific disease pathophysiology, these modules more likely represent a shared response pattern to diverse forms of brain injury, consistent with their overlaps with expression signatures from heterogeneous mouse disease models and aging. In contrast, other modules show patterns of overlap with mouse AD models that are strikingly divergent from those of the AD consensus clusters. For example, although CD12 strongly overlaps with cluster B, the overlaps with mouse models are more sparse than those seen in CD11. As the modules from Gandal et al. (2018) are much smaller than the AD consensus modules, they may represent subnetworks within the larger consensus clusters that are less strongly activated in AD mouse models.

### Non-obvious Mouse Models of AD

Our analyses also included many additional mouse genetic manipulations relevant to neurodegenerative mechanisms (Figure 2). Indeed, several non-AD mouse models showed overlaps with human AD modules mimicking those of canonical AD mice (Figure 5). For example, the C57BL6/J (B6J)-*nmf205* mouse (M182) overlaps significantly with modules from consensus clusters B and C (FPturquoise, p_adj_ = 6.4 × 10^−6^, and PHGbrown, p_adj_ = 9.6 × 10^−39^, respectively), behaving similarly to *MAPT* transgenics (M230, M243) and *CDK-P25* mice (M64, M65). The C57BL6/J (B6J)-*nmf205* mouse model has mutations in both the translation GTPase *GTPBP2* and the neuronal tRNA^Arg^_UCU_ genes that cause neurodegeneration through ribosomal stalling (Ishimura et al., 2014, 2016). In another example, expression of a mutant form of *neuroserpin* in mouse neural progenitor cells (Guadagno et al., 2017) activates a transcriptional signature (M205) that significantly overlapped with cluster A module IF-Gyellow (p_adj_ = 8.1 × 10^−17^) as well as FPblue (Figure 5B; p_adj_ = 4.7 × 10^−6^), a cluster D module showing some selectivity for AD transgenic models (above). Autosomal-dominant mutations in *neuroserpin* cause familial encephalopathy with Neuroserpin inclusion bodies, a rare, early-onset neurodegenerative dementia caused by protein aggregation within the endoplasmic reticulum (ER) and associated ER stress (Roussel et al., 2013).

Of the 30 human AD-associated coexpression modules, a minority showed virtually no overlap with AD mouse model brain expression signatures (Figure 4A, asterisk). These modules, largely corresponding to consensus cluster E, are strong candidates to represent features of AD pathobiology that are poorly recapitulated by existing AD mouse models. As discussed earlier, these modules showed comparatively poor enrichment for cell-type expression signatures (Figure 1B; Figure S2) and were similarly not well represented among curated AD pathways (Figure 1C), based on gene set enrichment analyses. Interestingly, our cross-species analyses highlight a number of other, unexpected mouse model expression signatures that overlap these modules (Figure 5). For example, conditional knockout of the DNA methyltransferase, *DNMT1* (M54), in the mouse brain (Narayanan et al., 2014) activates a set of DEGs that significantly overlapped (p_adj_ = 1.4 × 10^−11^) with a human module, IFGblue, enriched for unfolded protein response and DNA repair pathway genes. In another example, brain RNA-seq from a *Gnasxl*-deficient mouse (Holmes et al., 2016) (M156) overlapped with FPbrown, a coexpression module (p_adj_ = 5.4 × 10^−9^) enriched for genes involved in oxidative phosphorylation and mitochondrial translation. Interestingly, *Gnas*, which encodes a G-protein alpha stimulatory subunit, is a complex, imprinted genomic locus implicated in hypothalamic control of energy balance. Loss of the *Gnasxl* isoform causes a hypermetabolic mouse phenotype, resulting in growth retardation, hypoglycemia, and reduced adiposity (Nunn et al., 2013).

## DISCUSSION

We provide a systems-level molecular model of the AD transcriptional state in human brains. Our results highlight five dominant consensus clusters representing robust and reproducible patterns of coexpression patterns in brains affected by AD. These signatures are consistently observed across multiple cohorts and several brain regions and were identified by multiple independent coexpression algorithms. We further define correspondences between 30 human AD brain consensus gene expression networks and 251 mouse experimental comparisons, including models relevant to AD, other neurologic disorders, and aging. Overall, our meta-analysis of AD-associated gene dysregulation in human brains, along with the complementary cross-species comparisons in mouse models, provides a powerful resource to guide target selection and validation strategies.

Mouse genetic models have contributed enormously to our understanding of AD pathophysiology (Ballatore et al., 2007; Esquerda-Canals et al., 2017); however, the utility of these mice as robust preclinical models for AD has been challenged (Drummond and Wisniewski, 2017; Onos et al., 2016; Sasaguri et al., 2017). First, most AD mouse models are based on rare forms of familial autosomal-dominant AD, which are caused by single, highly penetrant gene mutations. In contrast, late-onset AD arises from dozens of other risk variants, including many with modest effect sizes (Karch et al., 2014; Kunkle et al., 2019), perhaps in combination with non-genetic risk factors. Second, unlike mouse models, most brain autopsies from individuals with AD show evidence of heterogeneous, mixed pathologies that likely modify disease onset, manifestations, and progression (Kapasi et al., 2017). Third, it has been suggested that widely used mouse behavioral assays may be poor predictors of clinically relevant outcomes in humans.

We find that many transgenic mice, including both *APP* and *MAPT* models, manifest gene expression signatures that significantly overlap with AD-associated coexpression modules from human brains. The most robust overlaps were detected among modules enriched for microglial and neuronal genes (clusters B and C). These findings are consistent with prior reports of similar expression signatures detected from human postmortem AD brain tissue (Conway et al., 2018; Grubman et al., 2019; Mathys et al., 2019; Mostafavi et al., 2018; Zhang et al., 2013) or from AD mouse models (Cummings et al., 2015; Gjoneska et al., 2015; Matarin et al., 2015). However, consensus clusters B and C account for only 14 of 30 coexpression modules (47%) and do not appear to be specific for AD pathophysiology. In contrast, a substantial minority of human AD coexpression modules had little to no detectable overlap with available AD mouse models. Among these, consensus cluster E includes signatures for certain inhibitory and excitatory cortical neuronal subtypes and was enriched for genes that regulate proteostasis. The overlaps we define highlight those molecular features of AD biology recapitulated by current mouse models. In contrast, non-overlapping modules may identify dimensions of AD pathophysiology that are poorly captured. We conclude that most AD mouse models show overall poor correspondence to human disease, based on brain transcriptomes, with the exception of neuronal and microglial-enriched coexpression modules. This is an important caveat for the interpretation of studies using these animal models and may explain in part their poor predictive power as preclinical models for AD. Our findings are consistent with a complementary study of mouse data from gene expression arrays (Hargis and Blalock, 2017).

Our analyses also highlight the value of experimental models for interpretation of human brain transcriptome profiles. Analyses considering either cross-sectional or longitudinal datasets similarly suggest that transcriptional changes overlapping human brain neuronal-enriched modules (cluster C) may represent an earlier, transient stage of AD. In contrast, our results suggest that cluster B modules, strongly enriched for microglial expression signatures, constitute a subsequent and more sustained AD endophenotype. Nevertheless, cluster B genes significantly overlap with candidate susceptibility loci from AD GWASs (Kunkle et al., 2019), which, along with other studies (Sala Frigerio et al., 2019), strongly suggests that although these changes may occur subsequent to disease onset, they are likely causal, perhaps affecting progression. Transition points between human-mouse overlaps can be linked to the manifestation of disease-relevant mouse phenotypes. For example, CRND8 *APP* mice reveal reduced synaptic markers and hippocampal neuronal loss at 6 months, when overlaps are first detected with cluster B modules (Adalbert et al., 2009; Brautigam et al., 2012). Similarly, in Tg4510 *MAPT* transgenics evaluated at 4 and 6 months, respectively, memory task impairment and neurodegenerative pathology correspond to sequential activation of neuronal and microglial expression patterns (Blackmore et al., 2017; Ramsden et al., 2005). As human brain RNA-seq can be evaluated only at the time of death, there are significant challenges to resolve age-dependent changes or to definitively establish links with clinical-pathologic progression. Collection of mouse RNA-seq from additional time points may therefore accelerate discovery of improved AD progression biomarkers and ultimately pinpoint critical windows for therapeutic interventions. Our cross-species approach also highlights the significant impact of sex on the AD brain transcriptome. Based on gene expression profiles, female AD mice progress more rapidly than males, and in a sex-stratified analysis of human brain gene expression, females demonstrated quantitatively greater transcriptional changes. Our results are consistent with prior observations in both mice (Jiao et al., 2016) and humans (Altmann et al., 2014; Li and Singh, 2014; Mayeux and Stern, 2012; Mathys et al., 2019; Sala Frigerio et al., 2019).

Aging is the strongest known AD risk factor. Using a distinct analytic design and largely independent datasets, Hargis and Blalock (2017) reported that DEGs were concordant between aging in humans and rodent models, a conclusion supported by our analysis. Strikingly, the majority of AD-associated human brain coexpression modules overlapping with *APP* and/or *MAPT* transgenic mouse models were also seen in aged, wild-type mice, as well as many other disease models. This result suggests that many human brain gene expression changes associated with AD, including neuronal- and microglial-enriched modules reported in other studies (Conway et al., 2018; Mostafavi et al., 2018; Zhang et al., 2013), may represent common transcriptional programs activated by the aging process itself. Rather than representing a specific signature of AD pathophysiology (e.g., Aβ- or tau-mediated mechanisms), these pathways appear to be activated by heterogeneous triggers, including those manipulated in mouse models of HD, ALS, SCA1, and other neurodegenerative disorders. Interestingly, several module overlap patterns still revealed possible disease-specific signatures. For example, several modules enriched for oligodendroglial markers (consensus cluster D) showed relatively specific overlap with AD mouse models, particularly APP transgenic models, and related human brain coexpression networks have previously been implicated in AD in multiple studies (Allen et al., 2018; McKenzie et al., 2017; Mostafavi et al., 2018). Alternatively, coincident activation of both neuronal- and microglial-enriched modules was seen preferentially in models characterized by significant tau pathologic burden. In contrast to AD and other neurodegenerative disease models, nearly all differential expression signatures from HD mice (35 of 37) (Langfelder et al., 2016) failed to overlap with microglial-enriched coexpression modules (Figure S5).

Our study has several notable limitations. First, as the overall correlation of the brain transcriptome and proteome is modest (Seyfried et al., 2017), it will be important in future work to consider whether human-mouse gene expression overlaps are improved at the protein level. Second, although our meta-analytic approach was designed to identify signatures of disease that are robust to technical and study-specific heterogeneity, it is possible that we could miss relevant gene sets with more modest coexpression or uneven representation across studies because of sample ascertainment differences. Furthermore, the resulting large size of the human consensus modules may limit sensitivity to detect significant overlaps using the hypergeometric test, especially for functional pathways represented by smaller gene sets. Compared with the laboratory preparation of mouse mRNA, the extraction and processing of human brain tissue is likely more susceptible to postmortem artifact, although our analyses adjusted for sample variability in postmortem interval. Moreover, the human RNA-seq data, along with the majority of included mouse studies, derive from bulk brain tissue, which includes mixed cell types. Indeed, most of the human coexpression modules are strongly enriched for cell type-specific signatures, which may therefore reflect global changes in cell proportions, such as neuronal loss or microgliosis. The growing availability of single-cell expression profiles can definitively address this concern. In addition, as the extent and tempo of neurodegeneration, including in both human and mouse models, can vary widely across different brain regions, RNA-seq profiles from whole brain might obscure more localized transcriptome overlaps. Last, although we selected nearly 100 independent mouse RNA-seq studies for inclusion in our analyses, prioritizing those most relevant to AD and neurodegeneration, we omitted many others with the potential to provide additional insights. In the future, our approach can thus be generalized to an even broader sample of available mouse data.

The strengths of our study include coexpression modules based on more than 2,000 human brain samples and consideration of a large and diverse number of mouse studies. All mouse RNA-seq data were reprocessed using a single, complementary pipeline to facilitate cross-species and cross-model comparisons. Importantly, unlike comparisons based on pathology or behavioral phenotypes, brain expression profiles likely represent more proximal endophenotypes, potentially affording greater sensitivity and reliability for detection of cross-species overlaps. In fact, we highlight several overlaps with transcriptomic endophenotypes from completely unexpected mouse experimental manipulations that manifest brain expression changes that mimic human AD and in some cases even overlap coexpression modules better than currently available AD mouse models. Such “AD transcriptologs” – mouse models based on transcriptome homology–may pinpoint non-obvious experimental models for future investigation of AD pathophysiology.

## STAR★METHODS

### RESOURCE AVAILABILITY

#### Lead Contact

Further information and requests for data or other resources should be directed to and will be fulfilled by the Lead Contact, Benjamin A. Logsdon (ben.logsdon@sagebionetworks.org).

#### Materials Availability

This study did not generate new unique reagents.

#### Data and code availability

All study data is available on the AMP-AD Knowledge Portal (https://doi.org/10.7303/syn2580853). The complete list of deposited data (with links), software, and relevant algorithms is provided in the Key Resources Table. Specifically, all original and re-processed RNA-seq data for the ROSMAP, MSBB, and Mayo clinic cohorts are available, along with the results of our differential expression meta-analysis across the three cohorts and 7 brain regions. Comprehensive results for coexpression analysis (2978 modules) generated using 5 algorithms across 7 brain regions in the three cohorts are also provided, along with the resulting aggregate AD coexpression modules (30 modules). Lastly, we also have deposited all data from the reprocessing of mouse model RNA-seq, along with the associated differential expression analysis, and complete results of mouse-human overlaps. An R package with all code for the metanetwork algorithm is available at https://github.com/Sage-Bionetworks/metanetwork, and a toolkit for integrating metanetwork with AWS high performance compute cluster cfncluster, and Synapse is also made available (https://github.com/Sage-Bionetworks/metanetworkSynapse). All code used to generate aggregate modules and figures are available in this R package: https://github.com/Sage-Bionetworks/AMPAD, with the following notebook collating the primary results: https://github.com/Sage-Bionetworks/AMPAD/blob/master/manuscript.Rmd.

### EXPERIMENTAL MODEL AND SUBJECT DETAILS

#### Human subject data

Details of sample collection, postmortem sample descriptions, tissue and RNA preparation, library preparation and sequencing, and sample QC are provided in previously published work (Allen et al., 2016; De Jager et al., 2018; Wang et al., 2018). Overall, the combined cohort consisted of 2,114 total samples from 1,234 subjects (Table 1; Table S1), including 478 AD cases, 300 controls (without AD pathology), and 456 subjects with progressive supranuclear palsy or other neurodegenerative pathologies. Sub-samples were selected to harmonize the late onset AD (LOAD) case - control definition across the three studies for all differential expression analyses. To compare analysis results across studies and to get an understanding of LOAD biology across different tissues, we harmonized the LOAD definition across three studies. The motivation was to define LOAD cases as those with both clinical and neuropathological evidence for definitive late onset Alzheimer’s disease - i.e., a high burden of neurofibrillary tangles, neuritic amyloid plaques, and cognitive impairment with little evidence of other pathology (Jack et al., 2018). Controls were concordantly defined as patients with a low burden of plaques and tangles, as well as no evidence of cognitive impairment if available. As such, for the ROSMAP study, we had individuals with a Braak neurofibrillary tangle (NFT) score (Braak et al., 2006) greater than or equal to 4, CERAD score less than or equal to 2, and a cognitive diagnosis of probable AD with no other causes as LOAD cases, Braak less than or equal to 3, CERAD score greater than or equal to 3, and cognitive diagnosis of ‘no cognitive impairment’ as LOAD controls. For MSBB, we analogously defined LOAD cases as those with CDR score greater than or equal to 1, Braak score greater than or equal to 4, and CERAD neuritic and cortical plaque score greater than or equal to 2 as LOAD cases, and CDR scores less than or equal to 0.5, Braak less than or equal to 3, and CERAD less than or equal to 1 as LOAD controls. It is to note here that the definitions of CERAD differs between ROSMAP and MSBB studies. For the Mayo Clinic RNASeq study, cases were defined based on neuropathology, with LOAD cases being based on Braak score greater than or equal to 4 and CERAD neuritic and cortical plaque score greater than 1 whereas LOAD controls being those defined as Braak less than or equal to 3, and CERAD less than 2. Further details concerning the diagnosis in the Mayo RNASeq study have been previously published (Allen et al., 2018). Summary of sample sizes of AD cases and controls are shown in Table 1.

#### Mouse Experimental Model Data

Figure 2 depicts the overall analysis pipeline for collecting and processing mouse studies, and examining overlaps with human coexpression modules. A total of 96 studies, encompassing data from 2279 mouse tissue samples, were analyzed (Table S3). These data were collected from three sources: the Gene Expression Omnibus (GEO) database (83 studies), the AMP-AD Knowledge portal (syn5550383) (6 studies), and through personal communication (7 studies). We searched the GEO database on September 12, 2017 using the keywords “brain,” “mouse,” and “expression profiling by high throughput sequencing,” identifying 881 studies for initial consideration. All studies were next indexed using high frequency terms, and secondary filtering was based on manually curated keywords relevant to AD pathophysiology (Karch et al., 2014) (Table S3). Lastly, the filtered list of 349 studies was reviewed by the study team. We excluded studies, or in some cases specific samples, involving (1) tissue source other than nervous system, (2) organisms other than mouse, or (3) non-coding RNA. From the remainder, 79 GEO studies were selected for inclusion based on relevance to AD, neurodegeneration, or related mechanisms. All included studies had publicly available RNA-seq data derived from either mouse brain or brain-derived cell lines. Following these initial searches, we discovered only a single eligible RNA-seq study for analysis of aging-associated expression changes (GSE61915). Given the importance of aging in AD, we identified and included an additional 5 expression array profiling studies related to brain aging. Table S4 details all studies included in this analysis, including data source, citations, and relevant keywords.

### METHOD DETAILS

As our study exclusively involves the analysis of previously generated data, all relevant further method details are described below.

#### Human RNA-Seq Reprocessing, library normalization and covariates adjustment

To avoid some of the technical variabilities arising due to RNA-seq alignment and quantification, and also to account for some of the technical variabilities we reprocessed and realigned all the RNA-Seq reads from the source studies (Allen et al., 2016; De Jager et al., 2018; Wang et al., 2018). The reprocessing was done using a consensus set of tools with only library type-specific parameters varying between pipelines. Picard (https://broadinstitute.github.io/picard/) was used to generate FASTQs from source BAMs. Generated FASTQ reads were aligned to the GENCODE24 (GRCh38) reference genome using STAR (Dobin et al., 2013) and gene counts were computed for each sample. To evaluate the quality of individual samples and to identify potentially important covariates for expression modeling, we computed two sets of metrics using the CollectAlignmentSummaryMetrics and CollectRnaSeqMetrics functions in Picard. To account for differences between samples, studies, experimental batch effects and unwanted RNA-Seq specific technical variations we performed library normalization and covariate adjustments for each study separately using fixed/mixed effects modeling. The workflow consists of the following steps: (i) gene filtering: Genes that are expressed more than 1 CPM (read Counts Per Million total reads) in at least 50% of samples in each tissue and diagnosis category was used for further analysis, (ii) conditional quantile normalization was applied to account for variations in gene length and GC content, (iii) sample outlier detection using principal component analysis and clustering, (iv) Covariates identification and adjustment, where confidence of sampling abundance were estimated using a weighted linear model using voom-limma package in bioconductor (Ritchie et al., 2015). For most analyses, we perform a variant of fixed/mixed effect linear regression as shown here: gene expression ~Diagnosis + Sex + covariates + (1| Donor) or gene expression ~Diagnosis x Sex + covariates + (1|Donor), where each gene is linearly regressed independently with Diagnosis, a variable explaining the AD status of an individual, identified covariates, and donor information as a random effect. Observation weights (if any) were calculated using the voom-limma (Ritchie et al., 2015) pipeline such that observations with higher presumed precision will be upweighted in the linear model fitting process. All workflows were applied separately for each of the three studies.

#### Network Inference and Module Identification

We apply 5 distinct network module identification methodologies to each of the 7 tissue specific expression datasets. This includes MEGENA (Song and Zhang, 2015), WINA (Wang et al., 2016), metanetwork, rWGCNA (Parikshak et al., 2016), speakEasy (Gaiteri et al., 2015), and a novel “metanetwork” algorithm to characterize a comprehensive landscape of transcriptomic variation across the seven brain regions and three studies. Briefly, MEGENA (Song and Zhang, 2015) is a method that infers a sparse graph based on a distance to define multiscale module definitions from coexpression data. Speakeasy is a label propagation method to identify robust coexpression modules that are identified both top up and bottom down (Gaiteri et al., 2015), rWGCNA is a version of WGCNA (Langfelder and Horvath, 2008) that includes bootstrapping to identify robust modules, WINA is also a variation on WGCNA that includes a modified tree cutting method to identify modules (Wang et al., 2016).

The metanetwork inference methodology is inspired by the DREAM5 method (Marbach et al., 2012), where ensemble inference methodologies were identified as more robust for identification of gene-gene interactions from coexpression data. We constructed a statistical network of gene co-expression using an ensemble network inference algorithm. Briefly, we apply nine distinct gene co-expression network inference methodologies ARACNE (Margolin et al., 2006a), Genie3 (Huynh-Thu et al., 2010), Tigress (Haury et al., 2012), Sparrow (Logsdon et al., 2015), Lasso (Krämer et al., 2009), Ridge (Krämer et al., 2009), mrnet (Meyer et al., 2007), c3net (Altay and Emmert-Streib, 2010) and WGCNA (Langfelder and Horvath, 2008) and rank the edge lists from each method based on the method specific edge weights, identify a mean rank for each edge across methods, then identify the total number of edges supported by the data with Bayesian Information Criterion for local neighborhood selection with linear regression. We identify metanetwork modules in each tissue type based on the inferred network topology with a consensus clustering algorithm (Wilkerson and Hayes, 2010) applied to multiple individual module identification methods. We ran individual network clustering methods applied to each of the seven network topologies. These methods included CFinder (Adamcsek et al., 2006), GANXiS (Gaiteri et al., 2015), a fast greedy algorithm (Clauset et al., 2004), InfoMap (Rosvall and Bergstrom, 2008), LinkCommunities (Ahn et al., 2010), Louvain (Blondel et al., 2008), Spinglass (Traag and Bruggeman, 2009), and Walktrap (Pons and Latapy, 2005), methods. All implementations are from the igraph package (Csardi et al.) in R.

#### Aggregate human coexpression module identification

For all 2978 modules identified across tissues, we first identify which modules are enriched for > = 1 AD specific differential expressed gene set from the DEG meta-analysis (see below). This restricts the total number of individual modules to 660 that show evidence of differential expression as a function of disease status. Next, we construct a within tissue module graph using a Fisher’s exact test for pairwise overlap of gene sets between each pair of these 660 individual modules. We then apply the edge betweenness graph clustering method (Pons and Latapy, 2005) to identify aggregate modules from these module graphs that represent meta modules that are both differentially expressed and identified by multiple independent module identification algorithms. With this approach we identify 30 aggregate module definitions across the seven tissue types and three studies (see Data and code availability).

In order to test the cross-region and cross-study robustness of these AD associated consensus clusters, we built aggregate modules from all of the individual modules generated for each brain region then compared these modules to the AD associated aggregate modules (Figure S2A). We find that there is significant overlap between the AD associated aggregate modules (AD) and the modules constructed with all individual modules (All) into the five consensus clusters. Those modules non-associated with AD do not show similar conservation across brain region, increasing confidence that the AD-associated modules are robust to technical and study artifacts.

#### Mouse RNA-seq re-processing

A unified RNA-seq analysis pipeline was used for reprocessing of all datasets, with the exception of 2 HD studies where count files were downloaded directly from GEO. Data processing leveraged the cloud formation cluster at Amazon Web Services. We first created one EC2 master instance (m3.xlarge) which was used to launch hundreds of EC2 computing nodes (c3.8xlarge). Next, each computing node was assigned to process one sample using our customized RNA-seq pipeline, as implemented using Snakemake v.4.8.0 (Köster and Rahmann, 2012). For samples in AMP-AD studies, the pipeline begins with downloading BAM files from the AMP-AD Knowledge Portal using the Synapse python client. The BAM files are then converted to fastq files using Picard SamToFastq command v.2.18.2 (http://broadinstitute.github.io/picard). For GEO studies, SRA files were downloaded from the database using the GEOquery R package v.2.42.0 (Davis and Meltzer, 2007), and fastq files were generated using the fastq-dump command from the NCBI SRA toolkit v2.8.2.1 (Andrews, 2010). Alignment to the mouse reference genome GRCm38 (mm10) was implemented using STAR v.2.5.1b (Dobin et al., 2013), and BAM file reads were subsequently sorted by coordinate using samtools v0.1.5 (Li et al., 2009). Genes were quantified using either HTSeq v0.6.0 (Anders et al., 2015) or using the ‘quantMode’ option from the STAR aligner which utilizes HTSeq algorithm and produces similar results. Results were uploaded to the Synapse portal using the python client.

#### Nomenclature for annotation of mouse differentially expressed gene sets

To facilitate ease of use and repurposing of the data, each mouse DEG set was assigned a unique identifier (M###), and we also developed a descriptive nomenclature for annotation. Each gene set received a label taking the form: *category_experimental condition_sex_age_brain region_cell type_ transgene.* In this standardized annotation, “category” denotes the relevant neurologic disease (e.g., AD, HD, SCA, ALS) or “other” for gene manipulations not directly linked to human disease, along with the specific “experimental condition” describing the mouse genotype or treatment condition. We also note “sex” (M or F), “age” (months), and where applicable, “brain region” (e.g., hippocampus), “cell type” (e.g., neuron, microglia). In the case of AD mouse models, we also annotate “transgene,” to differentiate “APP,” “Tau” (MAPT), or “other” models. If unknown or not applicable, the relevant field(s) are replaced with “na.” These annotations and conventions are used throughout our supplementary tables and files. The estimated pathologic burden (plaques/tangles and neuronal loss) in APP and MAPT models was annotated based on Alzforum (https://www.alzforum.org/research-models/) (Table S4).

### QUANTIFICATION AND STATISTICAL ANALYSIS

#### Meta-Differential expression analysis on human expression profiles

All the differential and meta-differential expression analysis were performed as weighted fixed/mixed effect linear models using the voom-limma (Ritchie et al., 2015) package in R. For each gene, linear regression was fit with biological and technical covariates that were associated with the top principal components of the expression data, as identified above. Two of the three studies - MSBB and Mayo RNaseq - obtained more than one tissue from the same donors. Therefore, except the ROSMAP study, donor-specific effects were explicitly modeled as random effects. Different models were built for understanding the effects of diagnosis and sex-specific diagnosis effects. Depending on the model, coefficients related to either diagnosis or diagnosis times sex was statistically tested for being non-zero, implying an estimated effect for the primary variable of interest is above and beyond any other effect from the covariates. This test produces a t-statistic (then moderated in a Bayesian fashion) and corresponding p value. P values were then adjusted for multiple hypothesis testing using false discovery rate (FDR) estimation, and the differentially expressed genes were determined as those with an estimated FDR below, or at, 5% with a corresponding absolute expression and fold-change cutoffs. To identify genes with evidence for change in expression across studies, we next performed a meta-analysis using a random effect and fixed effect models using rmeta r package (https://cran.r-project.org/web/packages/rmeta/index.html). The random effect model was selected as a conservative approach to correct for variation across studies. Sample size and characteristics of data included in the differential expression analyses are detailed in Table 1.

#### Human coexpression module enrichment analysis

Aggregate modules were interpreted using functional and cell type enrichment analysis. We performed a battery of enrichment tests to understand biological functionality, including evaluating primary hypotheses previously implicated by genetic findings in AD research, performing exploratory analyses of a large number of gene sets, and performing enrichment for brain tissue specific cell types. Among gene sets, we considered curated AD genes from GeneCards (Safran et al., 2010), Panther (Mi et al., 2017), dbGaP (Tryka et al., 2014), IGAP (Lambert et al., 2013), OMIM (Amberger et al., 2015), Biocarta (Nishimura, 2001), Wikipathways (Kutmon et al., 2016), and KEGG (Kanehisa et al., 2017). We also examined cell type markers from a mouse bulk brain tissue RNaseq atlas (Zhang et al., 2014) and human single-cell RNaseq (Lake et al., 2018). AMP-AD specific gene sets were constructed by taking the union of gene set definitions reported in each of the following reports: RNA-binding protein modules (Johnson et al., 2018), oligodendroglial modules from MSSM (McKenzie et al., 2017), AD versus Control oligodendroglial modules in the Mayo RNaseq study (Allen et al., 2018), and Module 109 from the ROSMAP study (Mostafavi et al., 2018). Genes not measured in our data are filtered from the annotated gene sets. Annotated gene sets with less than 10% of genes expressed in our datasets were removed. Fisher’s exact test was used to test enrichment of each gene set with the annotated set. Resulting p values were corrected independently for each set using Benjamini-Hochberg method for significance testing, owing to the differences in their hypothesis. Gene sets that had a minimum overlap of at least 3 genes were considered for further interpretation.

#### Differential expression analysis of mouse RNA-seq/microarray

Differential gene expression analysis was conducted using DESeq2 v1.18.1 (Love et al., 2014). For the limited number of microarray studies, pre-processed intensities available from the series matrix files were downloaded from GEO and normalized using quantile normalization, followed by differential expression analysis using the limma package v3.4.2 (Ritchie et al., 2015). For each study (Tables S3 and S4), experimental and control pairs were manually curated. We required a minimum of n = 2 samples for each group (experimental and control); the average for all samples included in each comparison was n = 8.4 (range = 4-28 total samples). Overall, 376 mouse experimental comparisons were curated for computation of differentially expressed gene sets (DEGs), applying a false-discovery rate (FDR) threshold of 1% and minimum fold-change of 1.2. The t-distributed Stochastic Neighbor Embedding (t-SNE) algorithm (van der Maaten and Hinton, 2008) was applied to DEGs from all studies (logarithm-transformed fold-change), using the Rtsne function in R. We excluded all DEG sets from consideration consisting of fewer than 10 conserved mouse genes, resulting in 251 sets of DEGs for consideration in our subsequent analyses. All analysis was done using R v3.4.2, and Python v.2.7.12. Table S4 details all DEG sets meeting these criteria, including data sources, and also enumerates the sample size (n) for all comparisons.

#### Statistical analysis of mouse-human overlaps

Mouse orthologs for all human genes were extracted using the HCOP tool available from the HUGO Gene Nomenclature Committee (see Data and code availability). Due to wide diversity of potential AD mechanisms, along with the number and heterogeneity of mouse models included in our analyses, it is not possible to confidently select the specific human gene-ortholog pairs in most cases where human genes map to multiple mouse orthologs. Therefore, in our primary analysis, we considered all possible human-mouse ortholog pairs (Table S5 and all results reported in manuscript). In a secondary analysis (Table S6), we performed a sensitivity test by restricting to human-mouse gene ortholog pairs with 1:1 relationships. In cases with multiple homologs, we selected the highest-confidence mouse ortholog for each human gene based on HCOP. Using the hypergeometric test, we determined the significance of overlap between each of 251 mouse DEG sets (above) and the 30 human gene coexpression modules (mouse orthologs) using the phyper function in R. The Benjamini-Hochberg method was applied to adjust for multiple comparisons, using the p.adjust function. All p values reported in the text were adjusted in this manner. Overall, out of 1569 significant module-DEG set overlaps (p_adj_ < 0.01) detected in our primary analysis, 1306 (83%) are recovered by the more the stringent sensitivity test using 1:1 human-mouse ortholog mapping. Consistency is even higher among 1058 highly significant overlaps (p_adj_ < 0.00001) from our primary analysis, for which 1014 (95.8%) are recapitulated using the more stringent 1:1 ortholog definition. Overlap significance was visualized using heatmaps, implemented with pheatmap function in R, using Manhattan distance (for both rows and columns) and Ward clustering. In order to determine whether mouse-human overlapping genes also shared expression changes in the same direction, we computed the concordance score for each overlap. Specifically, the concordance score is the percentage of genes in the concordant direction weighted by the significance based on the hypergeometric test, which is computed as follows:

WeightedConc=−log10(padj)×(ConcUp+ConcDown),

where

$$\mathrm{ConcUp}=\frac{\#\mathrm{overlapped genes up in both mouse and human}}{\#\mathrm{overlapped genes up in mouse}},$$

$$\mathrm{ConcDown}=\frac{\#\mathrm{overlapped genes down in both mouse and human}}{\#\mathrm{overlapped genes down in mouse}}$$

The overlap was considered concordant when the weighted concordance is greater (or less) than half standard deviation from the median, and p_adj_ ≤ 0.01 for the up- or down- differential expression. All computation and calculations were carried out in the R language for statistical computing (version 3.3.0 - 3.5.1) and Python v.2.7.12.

## Supplementary Material

1

2

3

4

5

## Figures and Tables

**Table 1.** AD Cases and Controls Included in This Study

Study	Total Subjects	Tissue	AD		Control		Total Samples
			Female	Male	Female	Male	
Mayo	179	CBE	47	32	35	37	151
		TCX	49	31	35	36	151
MSSM	164	FP	63	27	23	22	135
		IFG	55	24	17	20	116
		PHG	47	18	18	20	103
		STG	57	28	20	17	122
ROSMAP	241	DLPFC	109	46	47	39	241
Total	584		318	160	148	152	778

Counts (n) of AD case and control subjects and derived samples used for differential expression analysis. CBE, cerebellum; TCX, temporal cortex; FP, frontal pole; IFG, inferior frontal gyrus; PHG, parahippocampal gyrus; STG, superior temporal gyurs; DLPFC, dorsolateral prefrontal cortex. See Table S1 for details of the 2,114 samples included in the coexpression network analyses.

**Table 2.** Differential Gene Expression Meta-Analysis Results

Sex	Meta-Analysis Model	Upregulated Genes	Downregulated Genes
All	fixed	2,355	2,130
	random	1,773	1,577
Females	fixed	3,114	2,847
	random	2,211	2,036
Males	fixed	1,450	1,210
	random	845	567

The total number of AD-associated up- or downregulated genes are noted (false discovery rate [FDR] < 0.05 and mean expression fold change > 0.2) from joint and sex-stratified meta-analysis.

**Table:** KEY RESOURCES TABLE

REAGENT or RESOURCE	SOURCE	IDENTIFIER
Deposited Data		
AMP-AD individual AD coexpression modules	This study	https://doi.org/10.7303/syn10309369.1
AMP-AD aggregate AD coexpression modules	This study	https://doi.org/10.7303/syn11932957.1
AMP-AD aggregate AD coexpression modules gene-set enrichment results	This study	https://doi.org/10.7303/syn11954640.1
Differential expression meta-analysis of reprocessed RNASeq data from AMP-AD (all 7 brain regions)	This study	https://doi.org/10.7303/syn11914606
The Religious Order Study and the Memory and Aging Project (ROSMAP) RNA-seq	De Jager et al., 2018	https://doi.org/10.7303/syn3388564
The Mount Sinai Brain Bank (MSBB) RNA-seq	Wang et al., 2018	https://doi.org/10.7303/syn3157743
The Mayo clinic RNA-seq	Allen et al., 2016	https://doi.org/10.7303/syn5550404
Mouse orthologs for human genes extracted using the HCOP tool	This study	https://doi.org/10.7303/syn17010253.1
The AD Cross Species study (mouse model RNA-seq reprocessing, differential expression analysis, and mouse-human overlaps).	This study	https://doi.org/10.7303/syn16779040
Software and Algorithms		
R language v3.3.0-3.5.1	The R Foundation for Statistical Computing	https://www.r-project.org/
Picard v2.18.2-2.2.4	Broad Institute	https://broadinstitute.github.io/picard/
STAR v2.5.1b	Dobin et al., 2013 (PMID: 23104886)	https://github.com/alexdobin/STAR
voom-limma	Ritchie et al., 2015 (PMID: 25605792)	https://bioconductor.org/packages/release/bioc/html/limma.html
rmeta	CRAN	https://cran.r-project.org/web/packages/rmeta/index.html
MEGENA	Song and Zhang, 2015 (PMID: 26618778)	https://cran.r-project.org/web/packages/MEGENA/index.html
WINA	Wang et al., 2016 (PMID: 27799057)	https://doi.org/10.7303/syn7221264.2
metanetwork	This study	https://github.com/Sage-Bionetworks/metanetwork
rWGCNA	Parikshak et al., 2016 (PMID: 27919067)	https://github.com/dhglab/Genome-wide-changes-in-lncRNA-alternative-splicing-and-cortical-patterning-in-autism.
speakEasy	Gaiteri et al., 2015 (PMID: 26549511)	http://www.cs.rpi.edu/~szymansk/SpeakEasy
igraph	Csardi and Nepusz, 2006. https://igraph.org.	https://cran.r-project.org/web/packages/igraph/index.html
Snakemake v4.8.0	Köster and Rahmann, 2012 (PMID: 22908215)	https://snakemake.readthedocs.io/en/stable
GEOquery R package v2.42.0	Davis and Meltzer, 2007 (PMID: 17496320)	https://bioconductor.org/packages/release/bioc/html/GEOquery.html
NCBI SRA toolkit v2.8.2.1	N/A	https://github.com/ncbi/sra-tools
samtools v0.1.5	Li et al., 2009 (PMID: 19505943)	http://www.htslib.org/
HTSeq v0.6.0	Anders et al., 2015 (PMID: 25260700)	https://htseq.readthedocs.io/en/master
DESeq2 v1.18.1	Love et al., 2014 PMID: 25516281	http://www.bioconductor.org/packages/release/bioc/html/DESeq2.html
Python v.2.7.12	N/A	https://www.python.org/
HGNC Comparison of Orthology Predictions (HCOP)	N/A	https://www.genenames.org/tools/hcop

## Highlights

- Analysis of 2,114 postmortem samples reveals the Alzheimer’s brain transcriptome
- Overlap with mouse models pinpoints age-, sex-, and pathology-induced changes
- Cross-species resource highlights conserved transcriptional networks in disease
