# Supplementary material for: Meta-Analysis of the Alzheimer’s Disease Human Brain Transcriptome and Functional Dissection in Mouse Models
Source: Cell Rep. Author manuscript; Available in PMC 2020 Aug 14. (PMC7428328; doi:10.1016/j.celrep.2020.107908)
Supplement: 1 [file NIHMS1612517-supplement-1.pdf]

Cell Reports, Volume *32*

Supplemental Information

Meta-Analysis of the Alzheimer's Disease Human Brain Transcriptome and Functional Dissection in Mouse Models

Ying-Wooi Wan, Rami Al-Ouran, Carl G. Mangleburg, Thanneer M. Perumal, Tom V. Lee, Katherine Allison, Vivek Swarup, Cory C. Funk, Chris Gaiteri, Mariet Allen, Minghui Wang, Sarah M. Neuner, Catherine C. Kaczorowski, Vivek M. Philip, Gareth R. Howell, Heidi Martini-Stoica, Hui Zheng, Hongkang Mei, Xiaoyan Zhong, Jungwoo Wren Kim, Valina L. Dawson, Ted M. Dawson, Ping-Chieh Pao, Li-Huei Tsai, Jean-Vianney Haure-Mirande, Michelle E. Ehrlich, Paramita Chakrabarty, Yona Levites, Xue Wang, Eric B. Dammer, Gyan Srivastava, Sumit Mukherjee, Solveig K. Sieberts, Larsson Omberg, Kristen D. Dang, James A. Eddy, Phil Snyder, Yooree Chae, Sandeep Amberkar, Wenbin Wei, Winston Hide, Christoph Preuss, Ayla Ergun, Phillip J. Ebert, David C. Airey, Sara Mostafavi, Lei Yu, Hans-Ulrich Klein, Accelerating Medicines Partnership-Alzheimer's Disease Consortium, Gregory W. Carter, David A. Collier, Todd E. Golde, Allan I. Levey, David A. Bennett, Karol Estrada, T. Matthew Townsend, Bin Zhang, Eric Schadt, Philip L. De Jager, Nathan D. Price, Nilüfer Ertekin-Taner, Zhandong Liu, Joshua M. Shulman, Lara M. Mangravite, and Benjamin A. Logsdon

SUPPLEMENTAL FIGURES

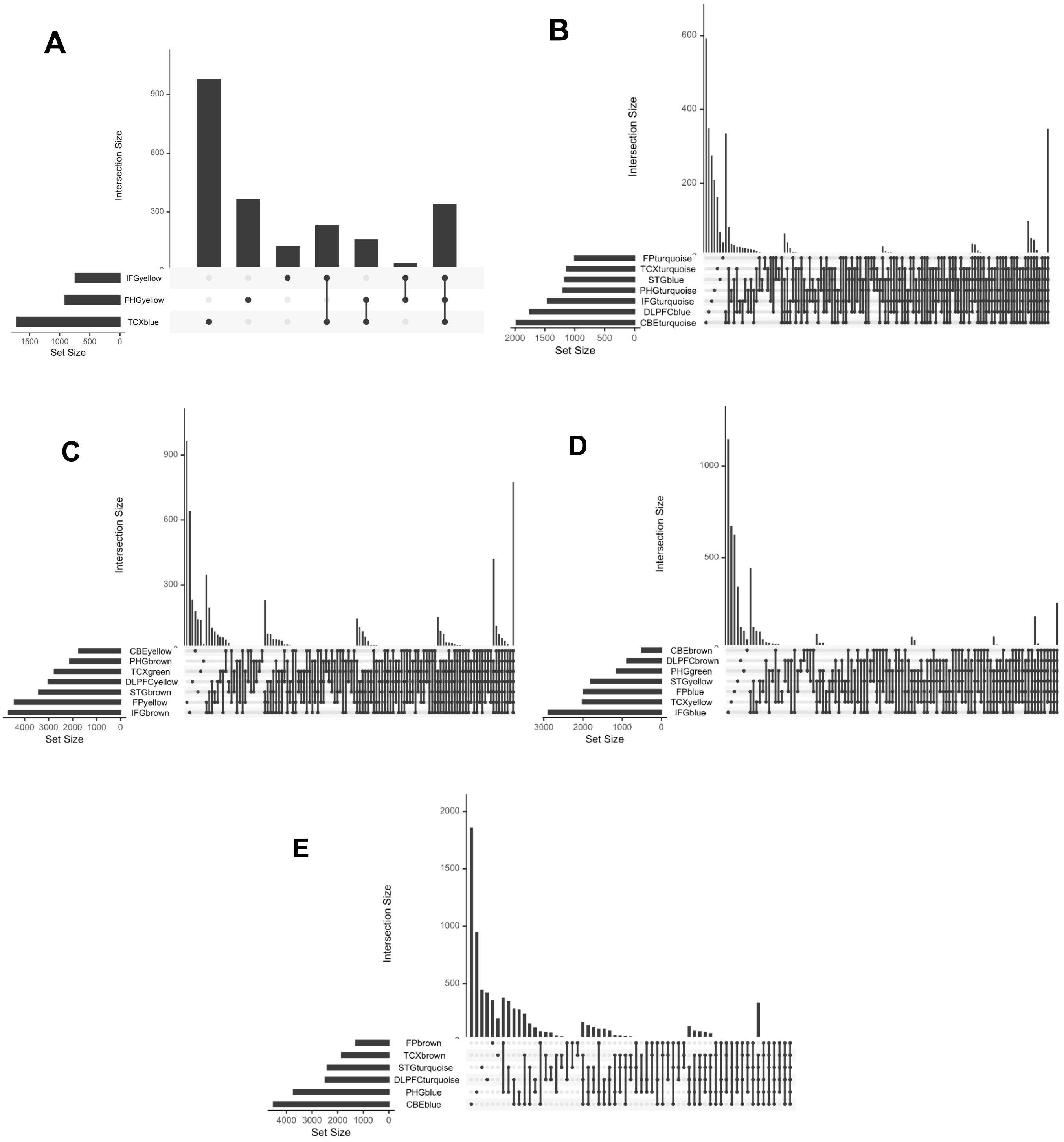

Figure S1: Human RNA-seq module sizes and gene memberships overlaps (Related to Figure 1).
UpSet plot of module size and module overlaps for consensus clusters **A** (TCXblue, PHGyellow, IFGyellow), **B** (FPturquoise, TCXturquoise, STGblue, PHGturquoise, IFGturquoise, DLPFCblue, CBEturquoise), **C** (CBEyellow, PHGbrown, TCXgreen, DLPFCyellow, STGbrown, FPyellow, IFGbrown), **D** (CBEbrown, DLPFCbrown, PHGgreen, STGyellow, FPblue, TCXyellow, IFGblue), and **E** (FPbrown, TCXbrown, STGturquoise, DLPFCturquoise, PHGblue, CBEblue).

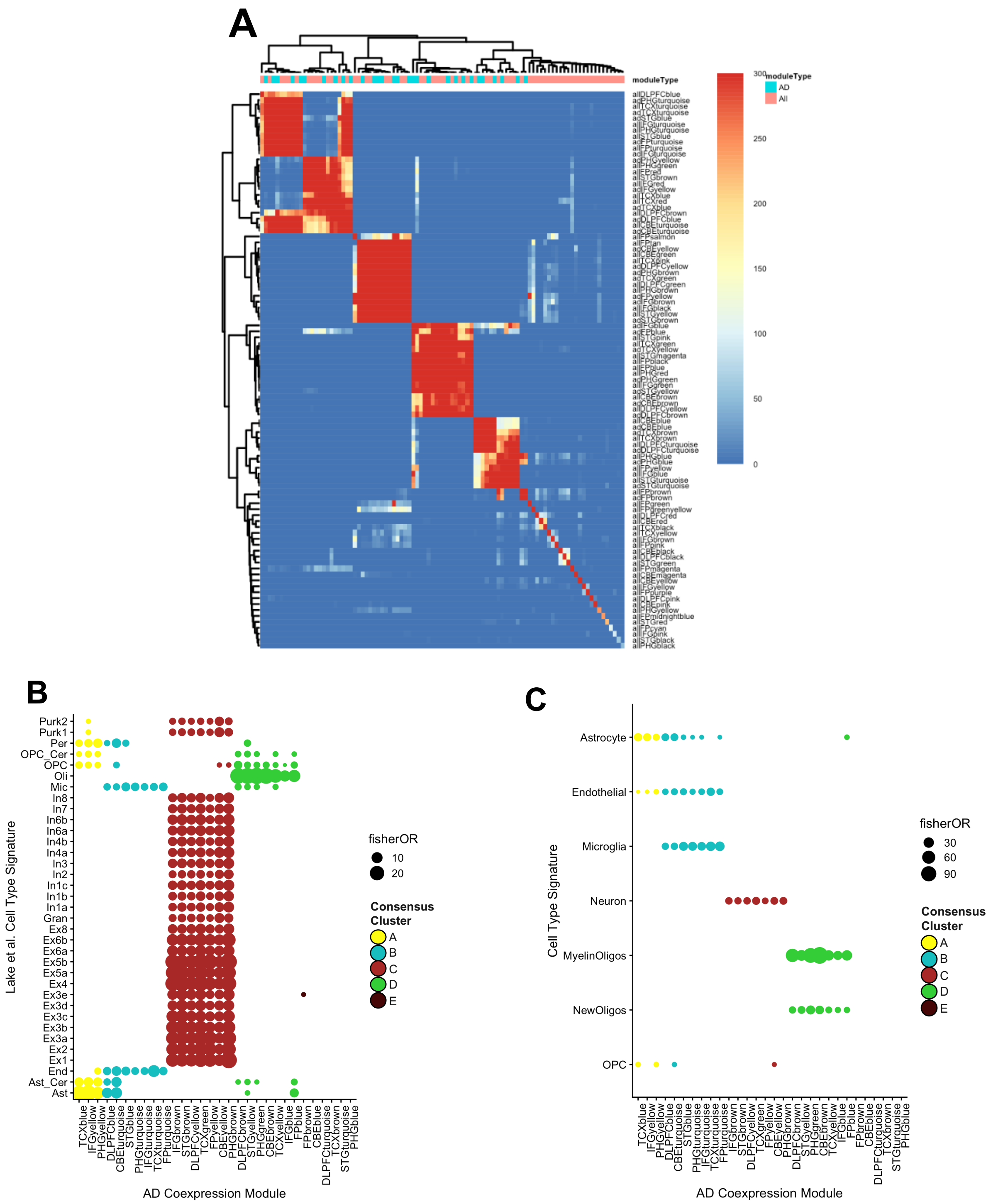

Figure S2: Module overlaps and cell-type specific enrichments (Related to Figure 1).
(**A**) Heat maps showing overlap between modules constructed using all individual coexpression modules (the individual modules are the modules built with the Megena, rWGCNA, metanetwork, SpeakEasy, and WINA methods) (All) compared to the modules reported in Figure 1 where we build modules only using the AD associated individual coexpression modules. (**B**) Cell type enrichments using Fisher's exact test of gene set overlap of the 30 AD associated coexpression modules with cell type specific gene sets from human reference single-cell RNA-seq data. (**C**) Cell type enrichments using Fisher's exact test of gene set overlap of the 30 AD associated coexpression modules with cell type specific gene sets from mouse reference RNA-seq data.

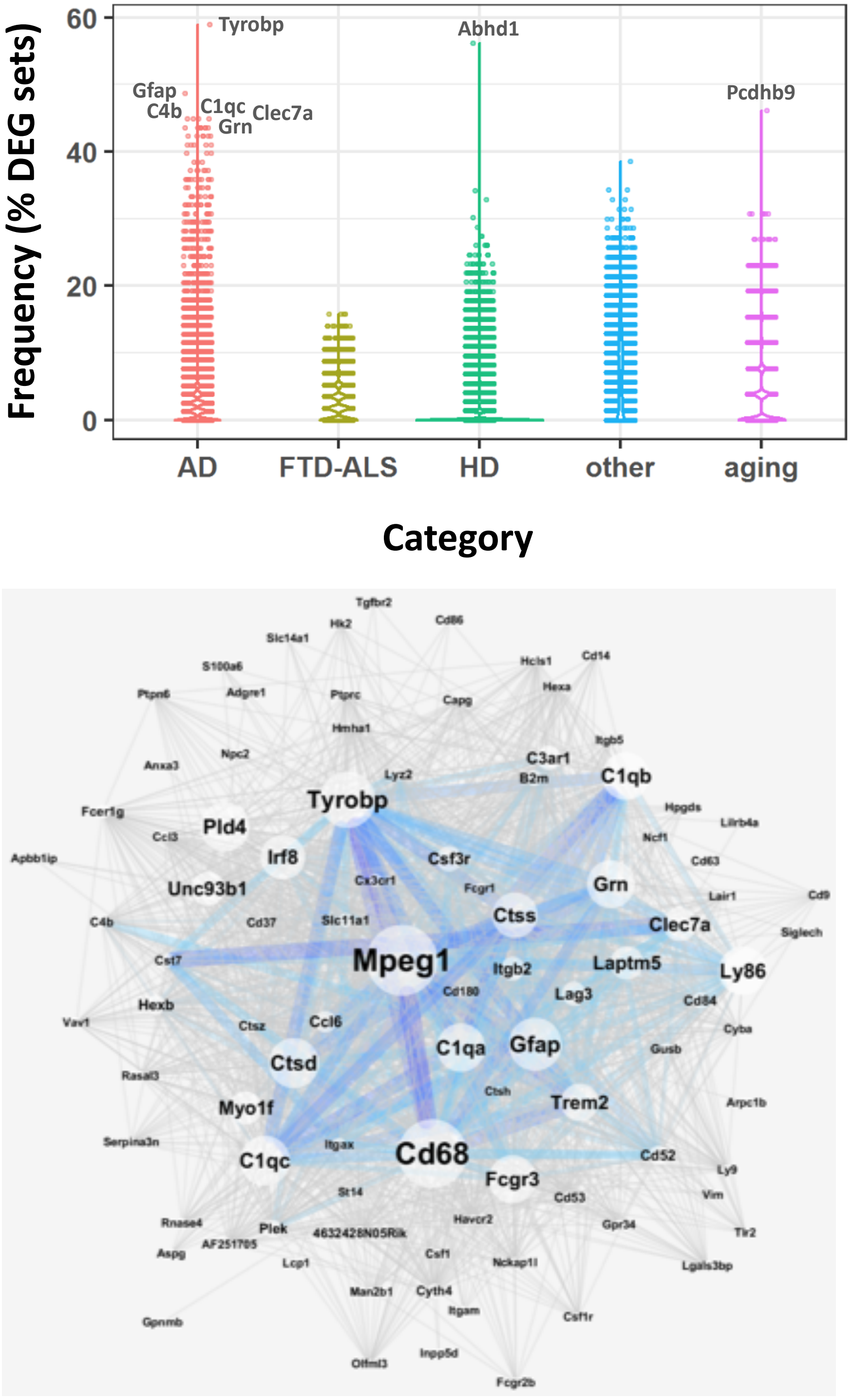

Figure S3: Mouse expression signatures are highly heterogeneous (Related to Figure 2).

(TOP) Recurrence rates for individual genes are shown among mouse DEG sets within each disease category. Most DEGs appear in only a minority of expression signatures, for a given disease category. Several highly recurrent genes among AD DEG sets are noted, several of which have roles in inflammation and innate immunity (see also Figure S3). (BOTTOM) Network depicting the most highly recurrent genes across 251 mouse model DEG sets and their co-occurrence relationships. Edges indicate co-occurrence of genes within a given model DEG set, with edge width indicating the number of times these genes recur together. The 95 genes depicted occur in at least 10% of the 376 DEG sets included in this study, and most significantly enriched for genes implicated in inflammation and immune system processes (GO:0002376, FDR=4.76x10^{-10}). Gene-pair co-occurrence counts were computed in R, and the network was constructed using the igraph package and visualized with Cytoscape. DAVID Bioinformatics Resources 6.8 functional tools was used to determine functional enrichment based on Gene Ontology (GO) terms.

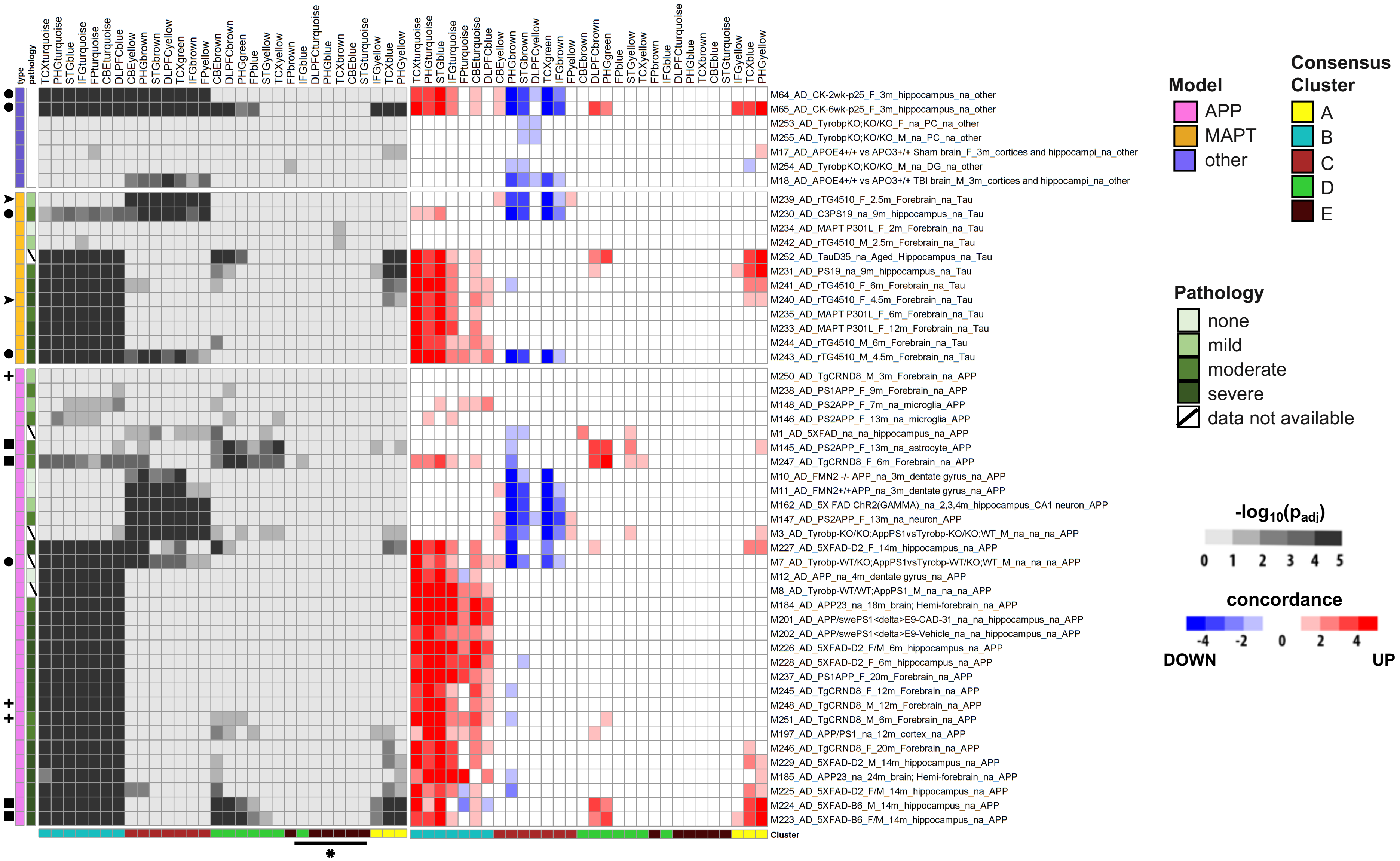

Figure S4: Human coexpression module overlaps with AD mouse models labeled with annotation (Related to Figure 4).
The data from Figure 4A is duplicated, but rotated and with full annotations of each model. Heat maps show overlap (left) and concordance (right) among 30 human coexpression modules and sets of differentially expressed genes (DEGs) derived from Alzheimer's disease (AD) mouse models.

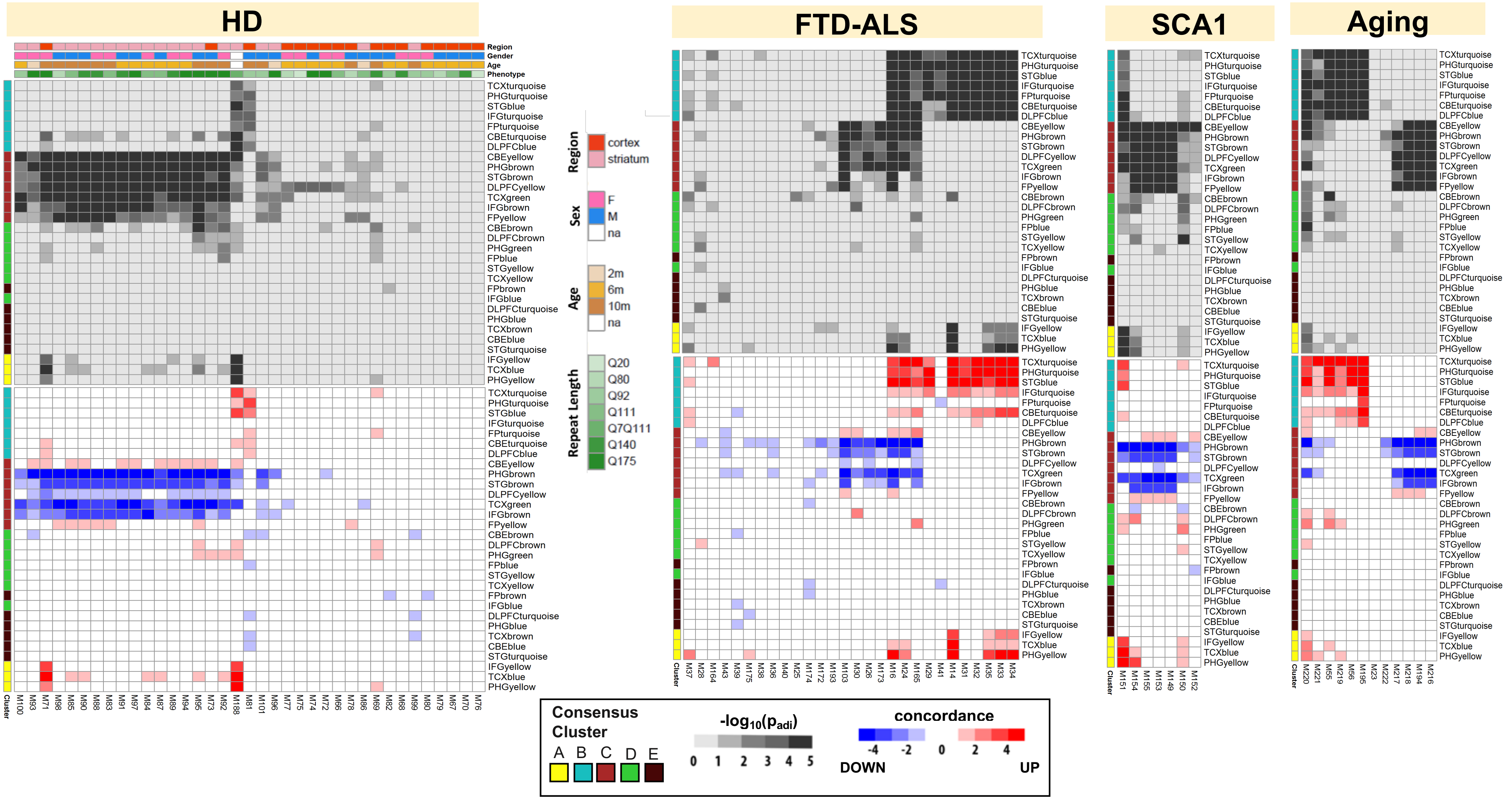

Figure S5: Human coexpression module overlaps with other disease models (Related to Figure 5).
Heat maps show overlap (top) and concordance (bottom) among 30 human coexpression modules and sets of differentially expressed genes (DEGs) derived from Huntington's disease (HD), frontotemporal dementia-amyotrophic lateral sclerosis (FTD-ALS), spinocerebellar ataxia, type 1 (SCA1), and aging mouse models. All HD DEG sets were derived from the same published resource, Langfelder et al., 2016. Mouse-human overlap significance, calculated using the hypergeometric test, is represented in grayscale [-log10(p_{adj})]. Annotation of direction (red/blue) and concordance (intensity) for mouse/human gene expression changes, as well as human module cell type enrichment following conventions in other figures.

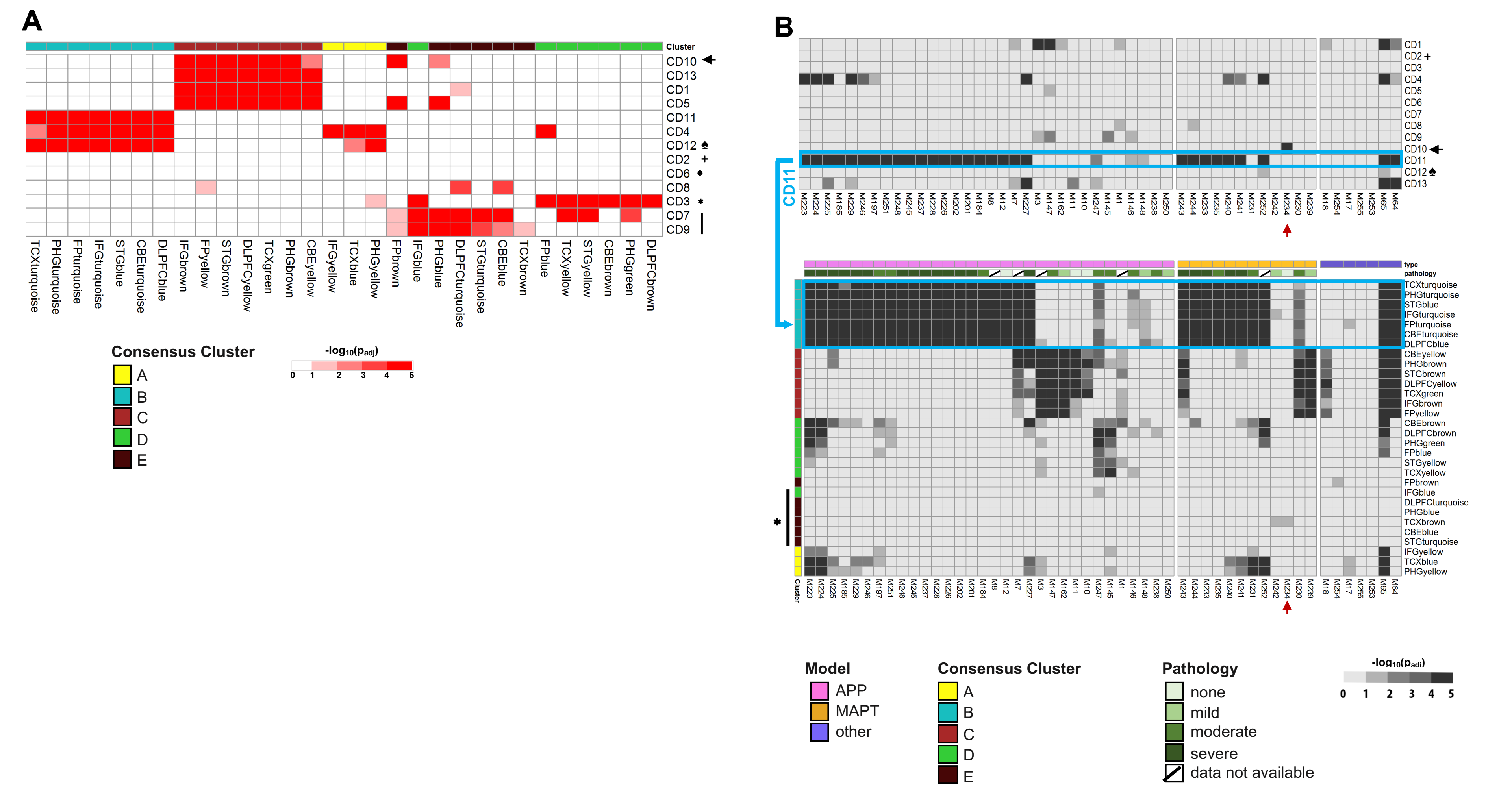

Figure S6: Overlaps with neuropsychiatric disease to assess AD specificity (Related to Figure 4).

Thirteen coexpression modules from Gandal et al. (2018) study were examined for overlap with (**A**) 30 AD-associated coexpression modules and (**B**) brain expression signatures from mouse models of AD (duplicated from Figure 4). (**A**) Eleven of 13 Gandal modules are associated with at least 1 of 5 neuropsychiatric disorders; CD3 and CD6 (asterisks) were non-associated. Several modules from clusters D and E are either entirely or predominantly AD-specific. For example, DLPFCbrown and CBEbrown only show substantial overlap with CD3, indicating that similar gene-expression changes were detected in the Gandal brain samples, but non-associated with any of the target disorders. STGyellow and DLPFCturquoise show restricted overlap with CD7 and/or CD9, which were associated with alcoholism (but not autism, major depression, bipolar, or schizophrenia). Other Gandal modules show strong overlaps with the AD consensus clusters, suggestive of non-specific gene expression changes. CD11 (associated with autism) and CD1 (commonly associated with 4 neuropsychiatric disorders) overlap with all modules in clusters B and C, respectively. As expected, CD11 overlaps with mouse models similar to the cluster B AD modules (Blue arrows and boxed region in panel B). Other Gandal modules (CD2, CD10, CD12) show divergent patterns of overlap with mouse AD models. Whereas CD12 and CD10 have significant overlap with clusters B and C (as with CD11 and CD1), the overlaps with mouse models are very sparse to non-existent (note black arrow and spade in panel B). CD10 only overlaps with M234 (red arrow) which is not overlapped by almost any AD consensus modules (weak overlap for TCXbrown only). CD2 (cross-hatch), associated with depression, fails to overlap any AD human consensus modules, nor does it overlap at all with AD mouse models.

SUPPLEMENTAL TABLES

Table S1. Data characteristics of the AMP-AD human RNA-Seq datasets (Related to Table 1).

Study	No of individuals	Tissue	RNA Library Prep	Strand	Sequencer	No of Samples	Median Read Depth (bn bases)
MAYO	302	TCX	Poly-A	Double	Ilumina HiSeq 2000	264	12.58
		CBE				263	13.5
MSSM	300	FP	Ribozero	Single	Ilumina HiSeq 2000	260	3.85
		STG				240	3.23
		PHG				225	3.56
		IFG				230	3.49
ROSMAP	632	DLPFC	Poly-A	Double	Ilumina HiSeq	632	8.36
**Total**	1234					2114	

Characteristics of the AMP-AD RNA-sequencing (RNA-Seq) data included in this study. Columns are as follows: Study = name of the study in which the RNA-Seq data was produced, No of Individuals = number of human subjects from which RNA-Seq data were obtained in each study, Tissue = the tissue sampled to obtain RNA-Seq data in each study (TCX = temporal cortex, CBE = cerebellum, FP = frontal pole, STG = superior temporal gyrus, PHG = parahippocampal gyrus, IFG = inferior frontal gyrus, DLPFC = dorsolateral prefrontal cortex), RNA Library Prep = method used to prepare RNA for sequencing (either positive poly-A selection or Ribozero ribosomal RNA elimination), Strand = strandedness of cDNA used for sequencing, Sequencer = model of sequencer used, No of Samples = number of samples from each brain region used for RNA-Seq, Median Read Depth (bn bases) = the median read depth for RNA-Seq data from each sampled brain region. Totals for the No of Individuals and No of Samples columns are provided in the bottom row of the table.

Table S2. Gene set enrichment results for gene sets involved in proteostasis (Related to Figure 1).

Module	heat shock transcriptional response[1]	Detection of unfolded protein[2]	Response to unfolded protein[3]	HSF1 activation[4]
CBEblue	13 (1.2x10^{-5})	8.4 (1.5x10^{-5})	8.4 (1.5x10^{-5})	5.9 (1.1x10^{-3})
DLPFCturquoise	8.5 (1.6x10^{-4})	9.1 (3.9x10^{-6})	9.1 (3.9x10^{-6})	5.4 (3.8x10^{-3})
TCXbrown	12 (7.6x10^{-6})	5.3 (4.4x10^{-3})	5.3 (4.4x10^{-3})	10.5 (6.1x10^{-6})
STGturquoise	11 (1.6x10^{-5})	8.1 (1.7x10^{-5})	8.1 (1.7x10^{-5})	4.8 (1.1x10^{-2})
PHGblue	9.5 (5.4x10^{-5})	3.6 (3x10^{-2})	3.6 (3x10^{-2})	7.6 (9.8x10^{-5})

Module-level breakdown of the enrichment of consensus cluster E for Gene Ontology (GO) terms involved in proteostasis. Module names are given in the first column, and all subsequent columns contain the description, and GO ID. GO term references are included in footnotes (below). Enrichment values are reported as fold-enrichment above expected values with the false discovery rate (FDR) reported in parentheses.

[1](Abravaya, Phillips and Morimoto, 1991; Fabregat *et al.*, 2016)

[2](GO:0002235) ('Gene Ontology Consortium: going forward', 2015)

[3]GO:0006986)('Gene Ontology Consortium: going forward', 2015)

[4](Zuo, Rungger and Voellmy, 1995; Cotto, Kline and Morimoto, 1996; Fabregat *et al.*, 2016)
